# Population structure of human gut bacteria in a diverse cohort from rural Tanzania and Botswana

**DOI:** 10.1186/s13059-018-1616-9

**Published:** 2019-01-22

**Authors:** Matthew E. B. Hansen, Meagan A. Rubel, Aubrey G. Bailey, Alessia Ranciaro, Simon R. Thompson, Michael C. Campbell, William Beggs, Jaanki R. Dave, Gaonyadiwe G. Mokone, Sununguko Wata Mpoloka, Thomas Nyambo, Christian Abnet, Stephen J. Chanock, Frederic D. Bushman, Sarah A. Tishkoff

**Affiliations:** 1 Department of Genetics, Perelman School of Medicine, University of Pennsylvania, Philadelphia, PA 19104 USA; 2 Department of Anthropology, School of Arts and Sciences, University of Pennsylvania, Philadelphia, PA 19104 USA; 3 Department of Microbiology, Perelman School of Medicine, University of Pennsylvania, Philadelphia, PA 19104 USA; 4 The Geisinger Commonwealth Medical College, Scranton, PA 18509 USA; 5 Department of Biomedical Sciences, University of Botswana School of Medicine, Gaborone, Botswana; 6 Department of Biological Sciences, University of Botswana, Gaborone, Botswana; 7 Department of Biochemistry, Muhimbili University of Health and Allied Sciences, Dar es Salaam, Tanzania; 8 Cancer Genomics Research Laboratory, Division of Cancer Epidemiology and Genetics, National Cancer Institute, Bethesda, MD 20892 USA; 9 Department of Biology, School of Arts and Sciences, University of Pennsylvania, Philadelphia, PA 19104 USA; 10 Present address: Kuopio Center for Gene and Cell Therapy, Microkatu 1, 70210 Kuopio, Finland; 11 Present address: Genomics England, Queen Mary University of London, London, EC1M 6BQ UK; 12 Present address: Department of Biology, Howard University, 415 College St. NW, Washington, DC USA

**Keywords:** Gut microbiome, Genetics, Diet, Adaptation, Sub-Saharan Africa, Hunter-gatherers, Pastoralists, Agropastoralists, Rural populations, Industrialization

## Abstract

**Background:**

Gut microbiota from individuals in rural, non-industrialized societies differ from those in individuals from industrialized societies. Here, we use 16S rRNA sequencing to survey the gut bacteria of seven non-industrialized populations from Tanzania and Botswana. These include populations practicing traditional hunter-gatherer, pastoralist, and agropastoralist subsistence lifestyles and a comparative urban cohort from the greater Philadelphia region.

**Results:**

We find that bacterial diversity per individual and within-population phylogenetic dissimilarity differs between Botswanan and Tanzanian populations, with Tanzania generally having higher diversity per individual and lower dissimilarity between individuals. Among subsistence groups, the gut bacteria of hunter-gatherers are phylogenetically distinct from both agropastoralists and pastoralists, but that of agropastoralists and pastoralists were not significantly different from each other. Nearly half of the Bantu-speaking agropastoralists from Botswana have gut bacteria that are very similar to the Philadelphian cohort. Based on imputed metagenomic content, US samples have a relative enrichment of genes found in pathways for degradation of several common industrial pollutants. Within two African populations, we find evidence that bacterial composition correlates with the genetic relatedness between individuals.

**Conclusions:**

Across the cohort, similarity in bacterial presence/absence compositions between people increases with both geographic proximity and genetic relatedness, while abundance weighted bacterial composition varies more significantly with geographic proximity than with genetic relatedness.

**Electronic supplementary material:**

The online version of this article (10.1186/s13059-018-1616-9) contains supplementary material, which is available to authorized users.

## Background

Gut microbiota have been shown to be affected by numerous factors, including host diet, medications, pets, socioeconomic status, environment of residence, and chance acquisition of lineages [1–15]. While temporary changes in diet have been shown to cause circumscribed shifts in gut bacterial composition, the dominant bacterial composition in healthy adults remains relatively stable and is influenced by long-term diet [12, 16–18]. Plant and animal domestication during the Neolithic period (~ 10 thousand years ago (kya)), and the shift from hunter-gatherer subsistence patterns to pastoralist and agriculturalist practices, constituted a major change in diet [19]. Numerous contemporary, rural African populations continue to practice traditional subsistence lifestyles, including pastoralism, hunting and gathering, and small-scale agropastoralism. Examining their microbiome composition and function can inform host-microbiota dynamics in the absence of the impact of industrialization and widespread antibiotic use.

Several cross-population studies have compared the gut microbiome of urban-industrial societies with traditional hunter-gatherer or agricultural societies. The latter two populations consume foods that are relatively lower in sugars, fats, and animal protein and are relatively higher in fiber [12, 20, 21]. The gut bacteria of urban-industrialized populations often have high abundances of *Bacteroides*, while the gut bacteria from traditional hunter-gatherer or agropastoral societies have higher abundances of *Prevotella* [22–28]. Whether these trends are due to the types or quantities of foods consumed, cultural or social practices, geographic, genetic, or other factors is unclear. Although there have been several studies of microbiome diversity within African populations [22–24, 27, 29–31], the range of gut microbiome compositions among African populations with diverse subsistence practices remains largely unknown.

**Fig. 1:**
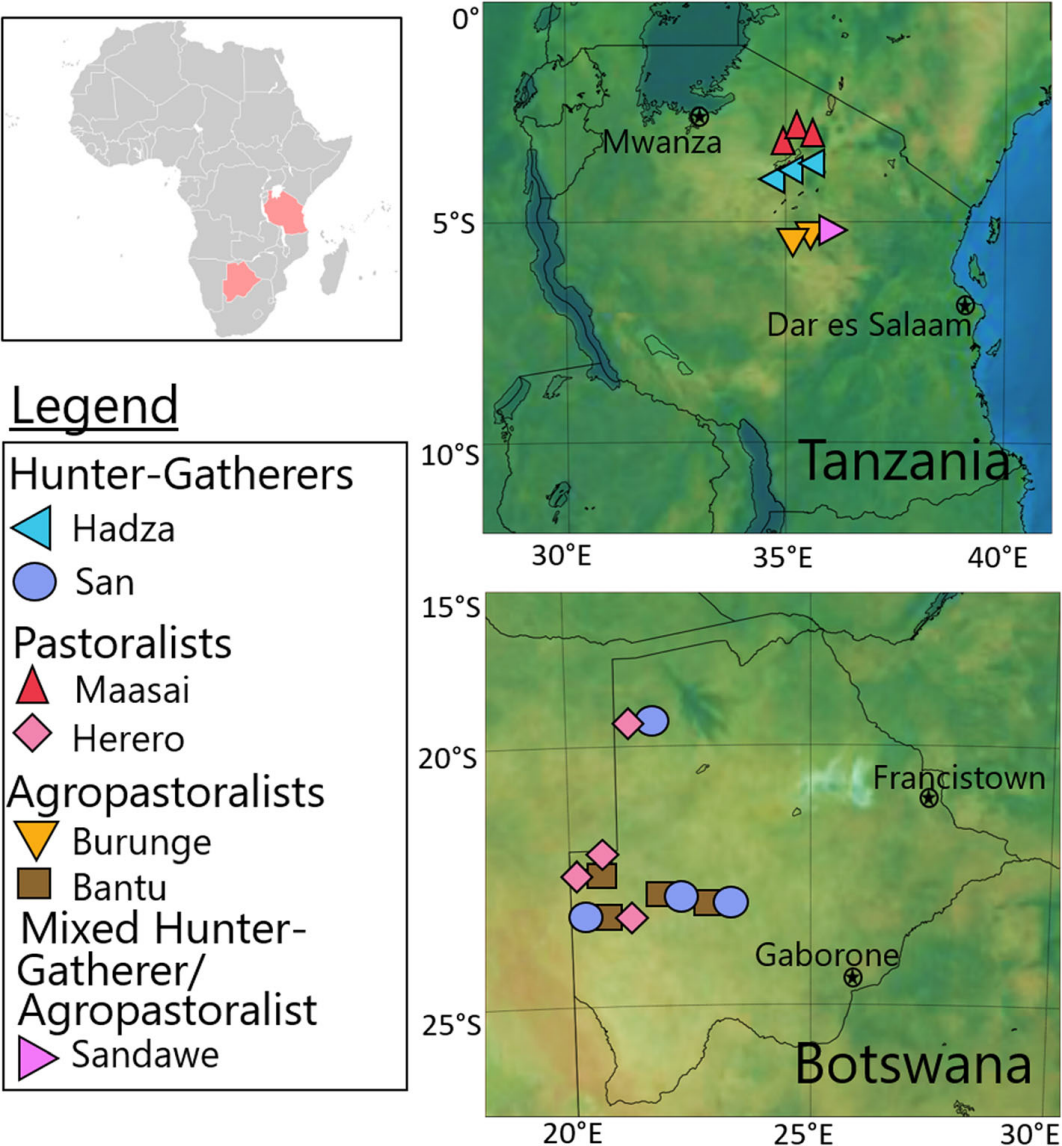
Map of the sampled population groups

Here, we present a comparison of gut microbiota from rural populations in Tanzania (*N* = 60), Botswana (*N* = 54), and individuals living in an urban US city (Philadelphia, PA) (*N* = 12) (Fig. 1, Table 1) [12, 32, 33]. The African populations are composed of multiple ethnic groups practicing varying degrees of hunting and gathering, agropastoralism, and pastoralism. “Pastoralists” are defined here as any population whose diet and economy are centered on cattle herding. We term populations whose diet and economy are centered around small-scale subsistence farming as “agropastoralists,” as every farming village we sampled also raised cattle or small livestock. Any population that derives most of its food from foraged plants and/or hunted game animals are termed “hunter-gatherers.”

**Table 1:** Cohort metadata per population group, listing country, population name, subsistence practice, number of individuals, and age range

Country	Population	Subsistence	Number (Total)	Number (Female)	Number (Male)	Age (Ave)	Age (Min)	Age (Max)
Tanzania	Burunge	Agropastoralist	11	10	1	48	22	70
	Sandawe	Agropastoralist	12	10	2	47.2	33	61
	Maasai	Pastoralist	12	6	6	39.5	24	68
	Hadza	Hunting and gathering	25	10	15	44.2	19	90
		Subtotals	60	36	24	44.5	19	90
Botswana	Bantu	Agropastoralist	26	19	7	49.8	24	92
	Herero	Pastoralist	8	7	1	44.5	19	77
	San	Hunting and gathering	20	15	5	28.0	18	42
		Subtotals	54	41	13	40.9	18	92
USA	Philadelphia	Industrial agropastoralist	12	4	8	26.2	22	33
		Totals	126	81	45	41.2	18	90

The four Tanzanian populations sampled are (1) the Khoesan click-speaking Hadza, who are savannah hunter-gatherers; (2) the Khoesan click-speaking Sandawe, who are former savannah hunter-gatherers that adopted agropastoral practices over a hundred years ago; (3) the Nilo-Saharan-speaking Maasai, who are semi-nomadic cattle herders; and (4) the Afroasiatic-speaking Burunge, who are agropastoralists. The three Botswanan groups sampled are (1) the Khoesan click-speaking San, who are hunter-gatherers of the Kalahari desert that have recently adopted some agropastoralist practices [34, 35]; (2) the Niger-Kordofanian Bantu-speaking Herero, who are Kalahari pastoralists; and (3) several groups of Niger-Kordofanian Bantu-speaking agropastoralists, hereafter referred to as “Bantu agropastoralists.” The US cohort is mainly composed of individuals who self-identified as “White,” with one self-identified “African American.”

## Results

### Data overview

DNA was extracted from stool samples, and the 16S rRNA gene V1-V2 segments were amplified and sequenced in all 126 participants. Sequences were aggregated at 97% identity, yielding 18,915 operational taxonomic units (OTUs). Seventeen thousand eight hundred seventy OTUs mapped to one of 191 bacterial taxa in the Greengenes classification database [36], 1044 OTUs were unassigned, and one OTU could only be mapped at the taxonomic resolution of Kingdom (Bacteria). The mean population abundance of unassigned reads was less than 0.15%, and we removed the 1044 unassigned OTUs and single Kingdom (Bacteria) OTU from further analysis (Additional file 1: Figure S1). Compared to the US samples, the African samples have a larger relative abundance of OTUs that were not confidently assigned to a known taxa (Additional file 1: Figure S1A). The four Tanzanian populations have the largest number of unassigned OTUs per individual (Additional file 1: Figure S1B), while the Sandawe have a larger number of total unassigned reads per individual compared to any other population (Additional file 1: Figure S1B). Collector’s curves showing the rate that new OTUs are detected as sample size is increased were calculated for OTUs with abundance > 0.01% and averaged per population (Additional file 1: Figure S2). These curves show that increasing our sample size would only marginally increase OTU counts. On average, the Sandawe have the highest number of OTUs, while the US have the lowest (Additional file 1: Figure S2).

### Abundance of *Prevotellaceae* varies within and between African populations

**Fig. 2:**
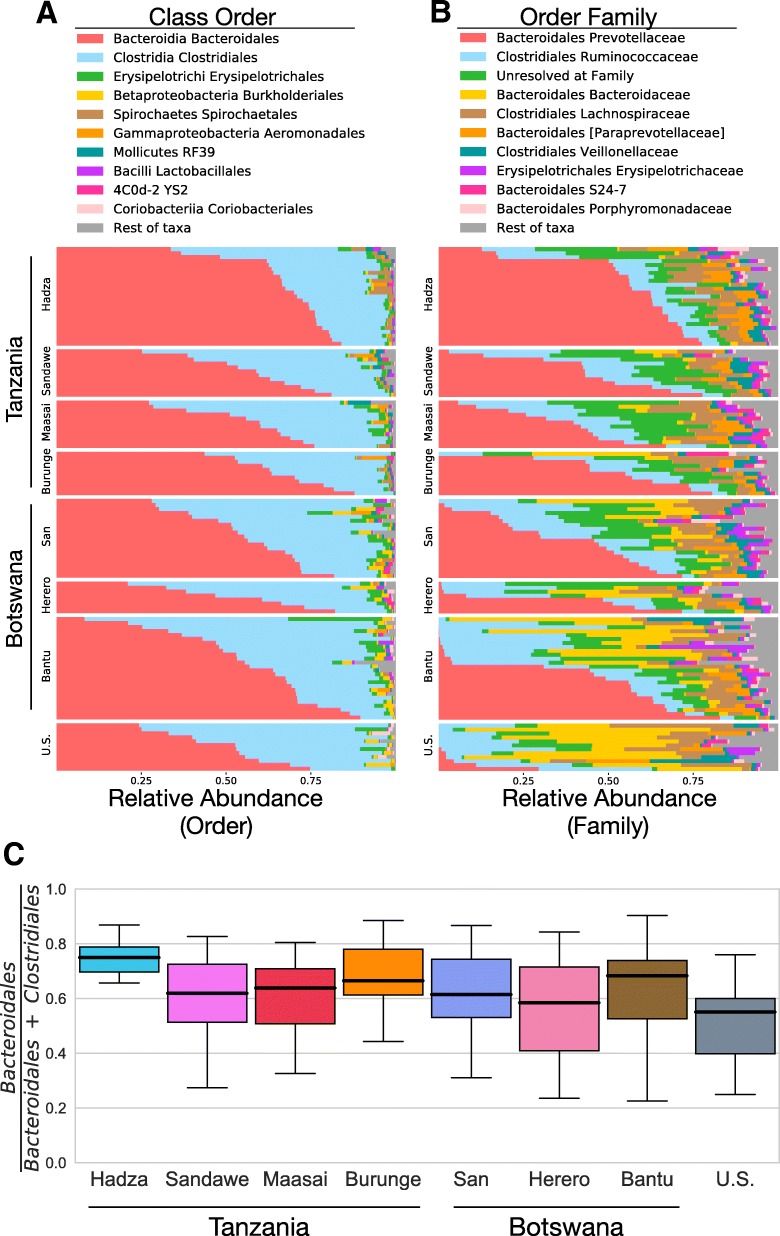
The relative abundance per individual for the ten most common taxa, shown for the bacterial taxonomic rank of **a** Order and **b** Family. **c** The population distribution of the relative proportion of *Bacteroidales* per total of *Bacteroidales* and *Clostridiales*

*Bacteroidales* (phylum *Bacteroidetes*) and *Clostridiales* (phylum *Firmicutes*) are the two most common orders of bacteria in nearly every individual (Fig. 2a), as expected for the human gut microbiome [37]. The relative proportions of *Bacteroidales* and *Clostridiales* varies by individual and by population (Fig. 2c). Comparing each population against the rest of the cohort and considering just the two taxa *Bacteroidales* and *Clostridiales*, we find that the Hadza have a significantly higher proportion of *Bacteroidales* (Mann-Whitney-Wilcoxon (MWW) test, *p* value 6.3 × 10^−4^), the US have a significantly lower proportion of *Bacteroidales* (MWW test, *p* value 0.020), whereas no other population had a significantly different proportion of *Bacteroidales* (smallest MWW test *p* value is 0.27).

*Prevotellaceae* is the most common bacterial family among the Africans in this cohort, being the most abundant family in 70.2% of Africans as well as having the largest mean abundance per population in every African population (Fig. 2b). Higher *Prevotellaceae* abundance has been previously associated with infection by the globally endemic gastrointestinal parasite *Entamoeba* in central African rainforest hunter-gatherers [30, 38]. Fecal DNA was screened for *E. histolytica* but this parasite was not detected in our samples, demonstrating that the high *Prevotellaceae* abundances are not due to *E. histolytica* infection in our samples. *Ruminococcaceae* is the second most common bacterial family in the African cohort, being the most abundant bacteria in 14% of Africans.

*Bacteroidaceae* is the most common bacterial family among the US cohort, being the most abundant family in 50% of US samples and having the largest population mean abundance. *Ruminococcaceae* is the second most common bacteria in the US cohort, being the most abundant bacteria in 25% of the US samples and having the second largest mean abundance.

Considerable variation in taxa abundances exists within African samples. In particular, 23 Africans have abundances of *Prevotellaceae* as low as what we find in the US samples (within one standard deviation of the mean US, an abundance of 12.4%). Among the African samples with such low *Prevotellaceae* abundance, 19 are from Botswana and of those, 12 are from the Bantu population. Fifty-two Africans were tested by quantitative PCR for absolute 16S rRNA copy numbers per gram of stool, including eight Bantu from Botswana. Of these eight Bantu, six individuals were in the low *Prevotellaceae* Bantu subset, and this group had the lowest average 16S rRNA copy number per gram of stool among any of the African groups (Additional file 1: Figure S3, Additional file 2: Table S1A, “Bantu_2”). We tested whether age, sex, host BMI, sampling latitude, or sampling longitude distinguished these 12 individuals from the other Bantu, but none were statistically significant (Wilcoxon rank sum tests, smallest *p* value is 0.41). Finally, we note that for 12 African individuals their most abundant bacterial family is not *Prevotellaceae*, *Ruminococcaceae*, or *Bacteroidaceae*, and in ten of these samples the taxa is unresolved at the level of family.

### Bacterial diversity per individual is higher in Tanzania than in Botswana

**Fig. 3:**
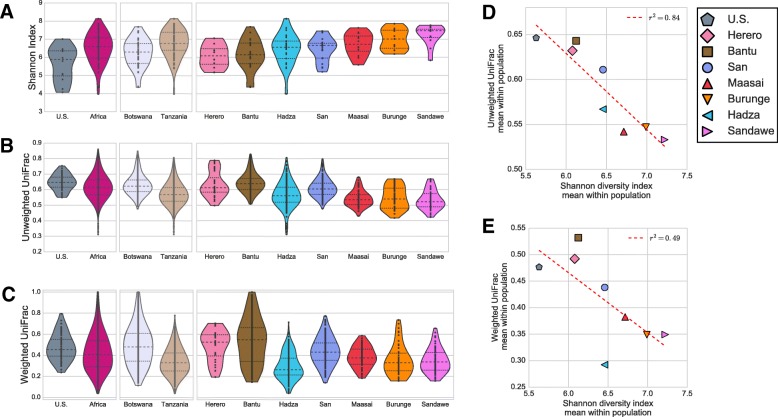
Within group mean *α* and *β* diversity. **a** Shannon index. **b** Unweighted UniFrac distance distribution within group. **c** Weighted UniFrac distance distribution within group. The within-population mean Shannon diversity versus unweighted and weighted UniFrac distances are shown in **d** and **e**, respectively

The African populations varied in gut microbial *α*-diversity (bacterial diversity, or bacterial richness and evenness, within each individual), as quantified with the Shannon diversity index (Fig. 3a). The US cohort had the least bacterial diversity, while the Sandawe had the highest, similar to previous comparisons of industrialized populations versus hunter-gatherers [24–27, 30] and small-scale agropastoralists [22, 23]. These trends are not impacted by rarefaction of OTU counts to 5000 per individual, as evidenced by the high correlation in Shannon diversity index with and without rarefaction (Spearman’s rho correlation 0.998, Additional file 1: Figure S4). *α*-diversity was not significantly correlated with the absolute 16S rRNA gene copy number (*R*^2^ = − 0.011, *p* value = 0.51) (Additional file 1: Figure S3E). However, we do find that the absolute 16S rRNA gene copy number is significantly higher in the Tanzanians than in the Botswanans (MWW test, FDR *q* value = 0.023) (Additional file 1: Figure S3D).

### Between host bacterial diversity is correlated with within-host bacterial diversity

The populations also varied in the within-population *β*-diversity (microbiota compositional dissimilarity between hosts), quantified by the UniFrac distance. The UniFrac distance is the fraction of the phylogenetic tree not shared between two samples, where the phylogeny of all taxa found in a bacterial community is estimated based on ribosomal RNA sequence similarity. Smaller values indicate greater sharing of the microbial phylogenetic tree among hosts within a population, which may reflect greater homogeneity in environmental factors (e.g., diet, cultural practices, shared geographic location). The Tanzanian Sandawe have the lowest within-population *β*-diversity, while the Botswanan Bantu and US have the largest within-population *β*-diversity (Fig. 3b, c).

We find a significant negative correlation between mean population *α*- diversity and mean within-population *β*-diversity for unweighted UniFrac distances (Fig. 3d, e) (unweighted UniFrac *β*-diversity: linear regression *R*^2^ = 0.84, *p* value = 1.47 × 10^−3^, and Kendall Tau correlation − 0.79, *p* value = 6.5 × 10^−3^; weighted UniFrac *β*-diversity: linear regression *R*^2^ = 0.49, *p* value = 0.052, and Kendall Tau correlation − 0.43, *p* value = 0.14). When individual pairs are restricted to the same sampling location for the within-population UniFrac calculation, the trend across Tanzanian populations is no longer evident, though the differences between Tanzania and Botswana remain (Additional file 1: Figure S5). The correlation between *α*- diversity and *β*-diversity also holds when counts are rarefied to 5000 reads per individual (Additional file 1: Figure S6), which accords with the high degree of correlation in UniFrac distances with and without rarefaction (Spearman’s rho correlation of 0.965 and 0.999 for unweighted and weighted UniFrac, respectively, Additional file 1: Figures S7 and S8). Additionally, the Bray-Curtis dissimilarity metric for beta-diversity yields similar results as the weighted UniFrac distance (see Additional file 1: Figure S9 and S10). Thus, the correlation between *α*- and *β*-diversity does not appear to be an artifact of choice of UniFrac as a *β*-diversity measure, uneven sampling location diversity, or uneven sequencing depth across individuals.

### Gut bacteria composition is more distinct between countries than between subsistence practices

The gut bacterial compositional differences between populations were quantified by the mean UniFrac distance between all pairs of individuals taken from between-population pairs. The between-population bacterial phylogenetic distances were larger between the US cohort and each African population than between any two African populations (Additional file 1: Figure S11, Additional file 2: Table S1B,C). The largest unweighted UniFrac distance within Africa was between the Botswanan Bantu and Tanzanian Hadza, which was nearly 92% of the average distance between the US and African populations. The largest weighted UniFrac distance within Africa was between the Bantu and the Herero in Botswana, which is nearly 84% of the mean distance average between the US and African populations.

**Fig. 4:**
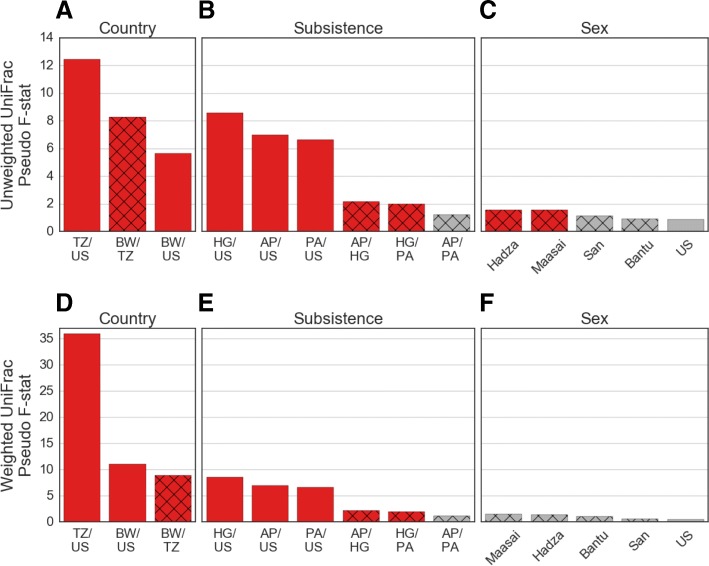
PERMANOVA tests of the phylogenetic difference between pairs of groups, based on unweighted UniFrac (panels **a**, **b**, **c**) and weighted UniFrac (panels **d**, **e**, **f**). Shown are groups defined by country of origin (panels **a**, **d**), subsistence practice (panels **b**, **e**), and sex (panels **c**, **f**). The subsistence practices are abbreviated as US = western (Philadelphian), HG = hunter-gatherers (Hadza, San), AP = agropastoralists (Bantu agropastoralists, Burunge, Sandawe), and PA = pastoralists (Herero, Maasai). Bar in red denote pairs where the *F*-statistic *p* value is < 0.05

The degree of compositional difference between two groups was assessed with PERMANOVA [39] tests of UniFrac distances, which measures the significance of the between-group variation to within-group variation (pseudo *F*-statistic) by permutation of group assignment. If two groups have identical distributions of bacterial composition, then the pseudo *F*-statistic will be ~ 1, with larger values corresponding to greater difference in composition between the two groups. As shown in Fig. 4, among pairs of countries, the USA and Tanzania have the largest pseudo *F*-statistic. The pseudo *F*-statistic for Tanzania and Botswana is as large, or nearly as large, as the pseudo *F*-statistic between the USA and Botswana (Fig. 4a, d, Additional file 2: Table S1D-G), which demonstrates that the bacterial compositional variation between two regional, rural, African cohorts can be of similar magnitude as the compositional variation between an urban/industrialized cohort and a rural African cohort.

Among the three pairs of African subsistence groups, the hunter-gatherers have significantly different compositions from both the agropastoralists and the pastoralists, while the agropastoralists and pastoralists are not significantly different from each other (Fig. 4b, e, Additional file 2: Table S1H,I). Comparing the magnitudes of difference between subsistence groups and geographic groups, we therefore find that the bacterial compositional difference between Tanzania and Botswana (a geographic grouping) is larger than between any of the African subsistence groups (both unweighted and weighted UniFrac *F*-statistics). From this observation, we infer that the gut bacteria are phylogenetically more distinct between groups defined by region (country) than by subsistence practice.

### Gut bacterial composition is significantly different between males and females in the Maasai and Hadza

Four populations were tested for differences between sex in bacterial *α*-diversity (Shannon diversity index, minimum of five individuals per sex for MWW test), Hadza, Maasai, San, and Bantu, and none showed a significant difference (all MWW test *p* values > 0.17). Five populations were tested for a significant *β*-diversity distance between sexes using PERMANOVA (UniFrac distances, minimum of four individuals per sex): Hadza, Maasai, San, Bantu, and US. The Hadza and Maasai had significantly larger unweighted UniFrac distances between sexes than expected by chance (PERMANOVA *p* value < 0.05) (Fig. 4c, Additional file 2: Table S1J), while no population had a significantly elevated weighted UniFrac distance between sexes (all PERMANOVA *p* values > 0.2, Fig. 4f, Additional file 2: Table S1K). Thus, there appears to be elevated phylogenetic differences between sexes in the Hadza and Maasai in terms of presence or absence of bacterial OTUs but not in terms of OTUs weighted by their abundance. Although we find a significant difference between sexes for these two populations, larger sample sizes will be needed to identify the factors causing these differences.

### Gut bacteria compositions of individuals from the US are more similar to Botswanans than to Tanzanians

**Fig. 5:**
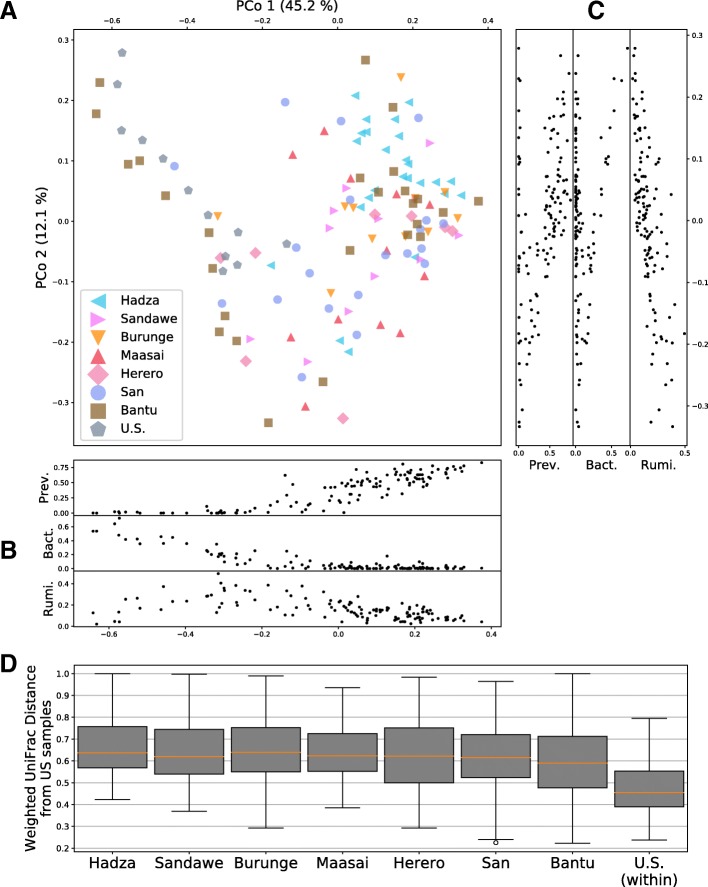
Principle coordinate analysis (PCoA) for weighted UniFrac distances. **a** The first two principle coordinates for all individuals in the study, where marker shape and color denote the population of origin. Sidebar (panels **b** and **c**) show the abundances of *Prevotellaceae* (Prev.), *Bacteroidaceae* (Bact.), and *Ruminococcaceae* (Rumi.) aligned to the first two principal coordinates. **d** Box-and-whisker distributions between each African population and the US samples, over all pairs of individuals

Using principal coordinate analysis (PCoA), we find that the similarities in overall bacterial OTU composition among individuals are strongly correlated with the abundances of three common bacterial families, *Prevotellaceae*, *Bacteroidaceae*, and *Ruminococcaceae* (Spearman’s rho correlation with PCo1 *p* values are 1.0 × 10^−40^, 1.0 × 10^−18^, and 3.3 × 10^−12^, respectively, and Spearman’s rho correlation with PCo2 *p* values are 5.0 × 10^−7^, 6.0 × 10^−2^, and 1.1 × 10^−20^, respectively) (Fig. 5, Additional file 1: Figure S12). The first principal coordinate (45% of variance) is most strongly associated with *Prevotellaceae* abundance while the second principal coordinate (12% of variance) is most strongly associated with *Ruminococcaceae* abundance.

The per population distribution of weighted UniFrac distances between Africans and the US cohort shows that the Botswanan gut bacteria are phylogenetically more similar to the US gut bacteria than the Tanzanian gut bacteria (Fig. 5d). This observation is consistent with the PERMANOVA results and the observation that there are more people with low *Prevotellaceae* abundance and high *Bacteroidaceae* abundance in Botswana than in Tanzania.

The 13 Bantu with high *Prevotellaceae* abundance similar to other Africans were markedly more different from the US cohort based on both weighted and unweighted bacterial composition as well as within-individual bacterial diversity (weighted UniFrac PERMANOVA test *p* value = 2.0 × 10^−5^; unweighted UniFrac PERMANOVA test *p* value = 2.0 × 10^−5^; MWW test on Shannon diversity *p* value = 0.014). By contrast, the 12 Bantu with low *Prevotellaceae* abundance, similar to the US cohort, were not statistically different from the US samples based on bacterial abundance (weighted UniFrac PERMANOVA test, *p* value = 0.12) but were different based on unweighted bacterial composition and within-individual bacterial diversity (unweighted UniFrac PERMANOVA test, *p* value = 3.0 × 10^−4^ and MWW test on Shannon diversities, *p* value = 0.028, respectively). Thus, the similarities between these Bantu and US individuals are driven by common bacteria.

### Observations of differentially abundant bacterial families among populations, subsistence groups, age, and sex

**Fig. 6:**
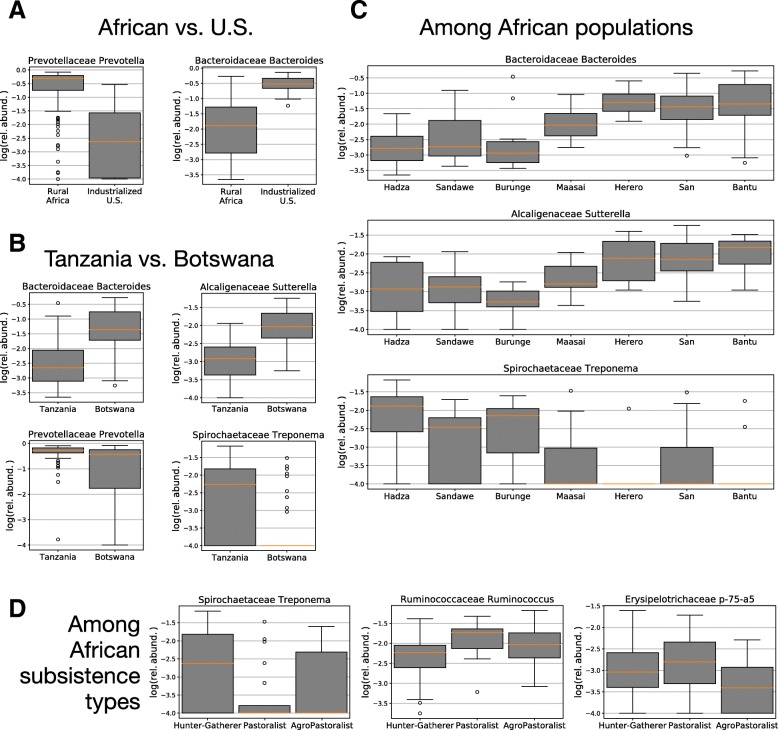
Box-and-whisker plots of relative abundances distributions per group for the taxa that varied significantly among groups by the ANCOM tests, where individuals are grouped by **a** traditional or industrial lifestyle, **b** country of origin, **c** population, and **d** traditional subsistence strategy

The analysis of composition of microbiomes (ANCOM) method [40] was used to test for significantly differentially abundant bacteria among groups defined by country, population, subsistence lifestyle, and sex. We found that two bacterial genera (out of *N*_genus_ = 48) vary significantly between Africa and the USA, *Bacteroides* and *Prevotella* (Fig. 6a), both of which also varied significantly between Tanzania and Botswana (Fig. 6b). We also observed that *Bacteroides* is one of several bacteria that are differentially abundant among the seven African populations (Fig. 6c). We note that 43.3% of all *Bacteroides* reads, and 35.2% of African *Bacteroides* reads, came from a single OTU (denovo36). Among the three African subsistence categories three genera varied significantly (Fig. 6d): *p-75-a5*, *Ruminococcus*, and *Treponema*. *p-75-a5* has previously been found in fecal samples from healthy children from Bangladesh [41] as well as in pre-weened calves [42]. *Ruminococcus* is also found in both human and ruminant fecal samples [43]. The fact that both *p-75-a5* and *Ruminococcus* bacteria have the highest abundance in pastoralists may be the result of close interaction between humans and livestock. The third genera that varies significantly among subsistence groups, *Treponema*, is most abundant in hunter-gatherers and agropastoralists, and has been previously associated with hunting and gathering and small-scale agropastoral populations with diets high in fiber [24, 25, 27]. Within the African cohort, no taxa were found to vary significantly between sexes nor among three age classes (18–39, 40–59, 60+).

### Imputed metagenomes show functional differences between populations and countries

The functional variation among populations was predicted using the metagenomic imputation method Phylogenetic Investigation of the Communities by Reconstruction of Unobserved States (PICRUSt) [44]. For every individual and Kyoto Encyclopedia of Genes and Genomes (KEGG) pathway, PICRUSt estimates the total gene count within that pathway (normalized to a relative abundance per pathway). Individuals were then grouped by population, subsistence, country, and continent, and statistical tests were computed on differences in the distribution of pathway abundances. One hundred forty-six KEGG pathways were significantly differentially enriched between the US and African cohorts, and 148 KEGG pathways were significantly differently enriched between Botswana and Tanzania (White’s nonparametric *t* test, FDR < 0.1) (Additional file 2: Table S1L-P). The pathway abundances of the Botswanan cohort are almost always intermediate between the Tanzanian and the US cohorts. The pathway relative abundance difference between Tanzania and Botswana is highly correlated with the pathway relative abundance difference between Africa and the USA (Spearman’s rho correlation 0.51, *p* value < 10^−22^; Additional file 1: Figure S13A). We infer that the regional differences in bacterial abundances may lead to regional differences in functional pathway abundances, depending upon the accuracy of gene content imputation. For example, we find that the degradation pathway of the pesticide dichlorodiphenyltrichloroethane, commonly known as “DDT,” is enriched in Botswanan samples but not in Tanzanian or US samples (Additional file 1: Figure S13B). No KEGG pathways varied significantly among African subsistence groups (ANOVA, FDR > 0.1).

Twenty-six KEGG pathways were significantly differentially enriched both between Africa and the USA and between Tanzania and Botswana and also have absolute relative differences > 15% between continent and between country (Additional file 2: Table S1L and Additional file 1: Figure S13A, shown in red). These are the pathways with the most extreme regional differences in pathway enrichment. Among these, five involve antibiotic biosynthesis or resistance, six involve the degradation of industrial xenobiotic compounds, nine involve digestion, and three involve cell recognition or cell-cell signaling. The frequencies for antibiotic resistance pathways and the xenobiotic degradation are highest in the USA and lowest in Tanzania, while the biosynthesis of the antibiotic ubiquinone has highest pathway frequency in Tanzania and lowest in the USA.

### Gut bacterial alpha-diversity is higher in people with low BMI

Previous research in humans and mice has observed correlations between lower *α*-diversity and prevalence of obesity [11, 45, 46]. Across all individuals (*N* = 126), we find that the *α*-diversity was significantly negatively correlated with the age- and sex-regressed BMI values (Kendall tau (KT) correlation − 0.21, *p* value = 6.2 × 10^−4^) (Additional file 2: Table S1Q), indicating lower bacterial diversity in individuals with higher BMI. The correlation remained significant among just the African individuals (*N* = 114, KT correlation − 0.18, *p* value = 5.1 × 10^−3^), although it was not significant within any single population (each population *p* value > 0.05). There is, however, a significant negative correlation within the pastoralists (KT correlation − 0.42, *p* value = 0.0094) and within the agropastoralists (KT correlation − 0.22, *p* value = 0.023). In addition, we tested for correlation (Kendall tau) between the regressed BMI values and the abundance of each bacteria at the taxonomic rank of genus with at least a 0.1% relative abundance in at least one population (*N*_genus_ = 56) (Additional file 2: Table S1R,S). We observed that 11 bacteria were significantly correlated with BMI (FDR < 0.01), the most significant of which are *Treponema* and *Anaerovibrio*.

To test whether the between-population differences in mean BMI (regressed on age and sex) drive the correlation between BMI and *α*-diversity over all individuals, we constructed “population re-centered” residuals by subtracting the population mean BMI from each individual’s BMI, according to their population of origin. The resulting correlation between the “population re-centered” BMI residuals and the *α*-diversity was not statistically significant (KT *p* value > 0.1 over all samples and over African samples only, see Additional file 2: Table S1Q). Similarly, none of the bacteria taxa at the rank of genus are significantly correlated with the population re-centered BMI residuals (FDR > 0.5). From this observation we conclude that the significance of the correlation between BMI and *α*-diversity is due to between-population differences. Thus, we cannot rule out that other host environmental or cultural covariates affecting BMI may be associated with bacterial diversity and abundance.

### Bacterial compositional similarity increases with geographic proximity and inter-individual relatedness

We investigated the differences in gut bacteria based on geographic distance and the degree of host genetic relatedness. A subset of 97 people was densely genotyped using the Illumina 5 M SNP array, with at least eight individuals from each African population, allowing for estimation of their genetic relatedness. To test whether genetic relatedness had any impact on the distribution of bacteria within a population, we calculated the correlation between host genetic relatedness and bacterial UniFrac distance among all pairs of individuals within each population. Genetic relatedness is quantified by the estimated identity-by-descent fraction, which is the fraction of the genome that is estimated to be identical between two people due to a shared recent common ancestor. Estimation of the identity-by-descent fraction assumes a panmictic population and is, therefore, reasonably suited for use as a within-population relatedness metric. To control for possible differences between sexes, we filtered the pairs of individuals to only include individuals of the same sex.

Only the Hadza have statistically significant correlations between identity-by-descent and both unweighted and weighted UniFrac bacterial distances (Additional file 2: Table S1T), while the Maasai have a significant correlation between identity-by-descent and unweighted UniFrac bacterial distance, indicating in both cases that more related individuals have more similar bacterial composition. Considering all tests, the correlation between identity-by-descent and unweighted UniFrac distance is negative in all but one case (unweighted UniFrac among the Herero). The probability that all seven weighted UniFrac correlations are negative by chance is < 0.01 (sign test), while the probability that at least 6 of 7 weighted UniFrac tests are negative by chance is 0.0625 (sign test). Thus, while we detect a statistically significant correlation between host relatedness and bacterial phylogenetic overlap only in the Hadza and the Maasai, there is a general trend for more related individuals to have more similar bacterial composition.

In addition, we examined the joint impact of geography and host relatedness on bacterial composition with a linear analysis of UniFrac distances. We modeled the bacterial phylogenetic distance (UniFrac) between hosts as a linear function of the host genetic relatedness and the host geographic separation: *U*_*ij*_~*D*_*ij*_ + *G*_*ij*_, where *i* and *j* are index individuals, *U*_*ij*_ is the bacterial UniFrac distance, *D*_*ij*_ is the geographic distance between the sampling sites for the individuals (measured in kilometers), and *G*_*ij*_ is the genetic relatedness of individuals. Here, we quantify *G*_*ij*_ with the correlation of normalized and centered genotype counts [47, 48]. This relatedness measure is widely used to control for population structure in cohorts drawn from multiple mating populations (e.g., genetic principal components analysis or as the covariance structure of random effects in linear-mixed models of genetic association tests) and, thus, is well suited as a measure of genetic relatedness when considering differences across genetically diverse populations.

The genetic relatedness and geographic distance between sampling sites are highly correlated (Spearman’s rho correlation − 0.66, *p* value < 10^−10^). We therefore regressed *G*_*ij*_ on *D*_*ij*_ and used the residuals, *G’*_*ij*_, when fitting the model *U*_*ij*_~*D*_*ij*_ + *G’*_*ij*_ to the observed data using linear least squares. For unweighted UniFrac bacterial distances, the best fit coefficients of *D*_*ij*_ and *G’*_*ij*_ are both significantly non-zero (*T* test *p* values < 0.002, Additional file 2: Table S1U), indicating that bacterial similarity is greater with closer geographic proximity and closer relatedness. For weighted bacterial UniFrac distances, only the coefficient of the geographic separation is significantly non-zero (*T* test *p* value < 0.001, Additional file 2: Table S1U). Although a linear model can only capture the main trends of the complex processes that shape the observed distribution of the gut microbiome, it serves to indicate that bacterial composition varies with geographic proximity and that the stratification with host relatedness is larger for bacterial presence/absence data than for abundance weighted data.

## Discussion

We surveyed the bacterial composition of fecal samples from rural populations in Tanzania and Botswana and a comparative population from Philadelphia in the USA. Among the rural Tanzanian and Botswanan populations, there are population level differences in bacterial diversity and abundances. We also found correlations between host BMI and both overall microbial diversity (less diverse microbiota were correlated with higher BMI) and the abundances of specific taxa. Host genetic similarity is correlated with more similar bacterial composition within the Hadza and Maasai populations. When comparing across African populations, we find genetic relatedness is correlated with presence/absence of gut bacteria, even when accounting for geographic separation.

The bacterial community diversity we observe between rural African populations is comparable to that observed by Gomez et al. [27] between two groups from the Central African Republic, the BaAka hunter-gatherers and a neighboring group of Bantu-speaking agriculturalists. The unweighted UniFrac distances between the BaAka and the neighboring Bantu is nearly 74% of the mean distance between the US and the African cohort, while for weighted UniFrac this ratio is nearly 70%. The African populations in our cohort are slightly more phylogenetically diverse based on unweighted UniFrac distances (83% of distance between USA and Africa) and are comparable in terms of weighted UniFrac distances (68% of distance between USA and Africa).

Given the difficulty of directly comparing microbiome studies that use different amplicons for OTU measurements, we contextualize our results with taxa-level meta-analysis from Smits et al. (2017), which identified four bacterial families and one bacterial phylum that primarily associate with traditional (*Prevotellaceae*, *Spirochaetaceae*, *Succinivibrionaceae*) or industrialized (*Bacteroidaceae*, *Verrucomicrobia*) populations (Additional file 1: Figures S14, S15; Additional file 2: Table S1V). Additionally, three of these five taxa (*Succinivirbionaceae*, *Spirochaetaceae*, and *Prevotellaceae)* were highly variable with season. With the inclusion of our study cohorts, this modified meta-analysis has bacterial compositional data from 26 populations in 17 countries (34 cohorts). For the US cohort used in this study, the mean abundances of the five taxa were within a standard deviation of the mean values for one or more US cohorts in the Human Microbiome Project (Additional file 1: Figures S14, S15; Additional file 2: Table S1V), indicating that it is not an outlier compared to prior studies.

The relative abundance of *Prevotellaceae* in the Hadza from our study was ~ 58%, which is nearly tenfold higher than the 8% relative abundance of *Prevotellaceae* found in the Hadza by Schnorr and colleagues [24], though it is within a standard deviation of the relative abundance reported by Smits et al. [31] (~ 38%) (Additional file 1: Figures S14, S15; Additional file 2: Table S1V). Prior studies of the Hadza (Smits et al. 2017) [31] and the Hutterites from the USA [49] indicate that seasonally volatile gut bacterial taxa correlate with seasonally available food. The Hadza in our study were sampled mid-October through early November, which is the late dry season and the beginning of the wet season, when there is an average rainfall of 57 mm [50]. Schnorr and colleagues sampled from the Hadza population in the rainy season during January [24], when average rainfall is ~ 146.6 mm [50]. The increased abundance of *Prevotellaceae* in the Hadza in our study is concordant with seasonal variation of this taxa reported in Smits et al. [31]. As with any bacterial taxa and study population, differences in *Prevotellaceae* abundance between microbiome studies of the Hadza could be affected by use of different protocols, reagents, and primers.

Fluctuations in short-term diet could also explain some of the variability seen between microbiome studies [51] in the Hadza and our other sampled populations. Although we were not able to get individual or population level dietary information for our research participants, we conducted a nutritional literature review to provide a qualitative assessment of contemporary diet in the traditional populations presented in this study (see Additional file 2: Table S1W and Methods for extended dietary information). Given the dissimilarity of food types between industrialized and traditional populations, the compositional similarity between the Bantu and US is noteworthy and may be reflective of individual nutritive changes in the Bantu from Botswana and a shift from traditional to industrialized diets. It is clear from Fig. 2 that there is heterogeneity in bacterial abundance profiles within the Bantu, where roughly half the population has gut bacteria similar to the other African groups, and the other half has gut bacteria more similar to the US cohort. We could not identify any host factors (age, sex, BMI, location) that significantly distinguish these two groups of Bantu. If we are observing a population undergoing changes in life styles that impact gut bacteria, then the changes in gut bacteria are not uniform across the population. Future work aimed at pairing longitudinal gut microbiome research with individual and population level dietary surveys would be informative for determining the extent to which shifts in subsistence and diet affect microbial changes.

*Bacteroides* has been used to distinguish between developing (low *Bacteroides* abundance) and industrialized (high *Bacteroides* abundance) populations [22–28, 30] and is significantly variable across the African populations, with generally higher abundances in Botswanans than in Tanzanians. We find that the gut bacterial composition of the US population is closer to the Botswanan populations than to any of the Tanzanian populations. In particular, the US gut bacterial composition was most similar to the Botswana Bantu agropastoralists, and 12 of the Botswana Bantu agropastoralist individuals have gut bacteria that are not significantly different from the US individuals by abundance weighted composition. The US and Botswana Bantu agropastoralists also have the two lowest measures of taxonomic diversity within hosts and two of the highest measures of inter-individual diversity in this cohort. Botswana is more economically developed than Tanzania, reflected in higher yearly per capita gross national income ($15.5 k in Botswana to $1.75 k in Tanzania), and a higher percentage of Botswanans (57%) than Tanzanians (30%) live in urban areas [52, 53]. None of the populations in our study live in an urban setting; sites are ~ 60 km or more walking distance from the nearest town (see Additional file 2: Table S1X for more details). It is possible that there are country-level differences in pathogens, sanitation, hygiene practices, transportation access, or medical access between Tanzania and Botswana that impact gut microbiome composition in rural areas.

The Hadza, San, and Sandawe are three current or former hunter-gatherer populations in various stages of settlement or transition from their ancestral subsistence lifestyle. The Sandawe settled into villages and adopted small-scale agropastoral practices in the mid-1800s [54]. The Sandawe have the greatest bacterial *α*-diversity in the cohort, which may be related to their genetic admixture with neighboring populations and/or their mix of subsistence practices. Varying subsistence strategies could plausibly increase gut bacterial diversity (1) neutrally, through the introduction of a wide array of microbes due to a varied life style and diet, or (2) selectively, due to bacterial community adaptation to varying environments. The high Shannon diversity values in the Sandawe are consistent with the “Intermediate Disturbance Hypothesis” which proposes diversity of bacteria is maximized under conditions of fluctuating environments (e.g., diet in this case) [55, 56].

The Hadza are unique in this cohort in that they still largely practice hunting and gathering [57]. Their gut bacteria are outliers in several respects: (A) they have the highest abundance of *Prevotellaceae* and *Spirochetaceae* [24–27, 30], in particular, the genus *Treponema* within family *Spirochetaceae*, which is a common constituent of hunter-gatherer gut microbiomes [24–27, 30] and a catabolizer of fibrous plant materials (cellulose and xylans) that form a large component of Hadza diets [24], (B) the Hadza are outliers in unweighted UniFrac PCoA, indicating that their bacteria, in terms of presence/absence, are phylogenetically the most dissimilar to other African populations, (C) they and the Maasai are the only two populations (out of five tested) with a significantly distinct microbiome between sexes, (D) the Hadza common gut bacteria are phylogenetically more homogenous across the population relative to all other populations in this study, and (E) the within-population variation in their gut bacteria is correlated with the relatedness among individuals, where more related individuals tend to have more similar bacterial composition (both presence/absence and abundance weighted). The bacterial phylogenetic differences between sexes in the Hadza that we observe corroborates a previous finding of sex differences in the Hadza gut microbiome [24], and may be partly attributable to sexual division of labor and differential food intake [24]. Hadza men and women have different activity patterns, where men are highly mobile foragers with access to honey and game meat, while Hadza women forage for local materials and may engage in more frequent “snacking” on fiber-enriched foods than men [58, 59].

The Maasai of Tanzania and the Herero of Botswana are two cattle herding peoples that live in close proximity to domesticated animals and have a heavy dairy component to their diet [60]. However, the Maasai and Herero gut bacteria are not more similar to each other than to other neighboring populations in their respective countries. The Maasai, like the Hadza, have a significant distinction in bacterial communities between sexes. Maasai men are in charge of supervising and herding cattle [60, 61] whereas women traditionally manage the household, oversee milk production from animals and milk distribution (or sale), and supervise small livestock (goats, sheep) [60, 61]. The separation of labor and the time away from home spent by men while tending cattle [62] could affect the types and quantities of food that men eat compared to women.

Across the seven African populations, we find a significant negative correlation between *α* and *β*-diversity, which corroborates a trend that has been previously observed between pairs of Western and non-Western populations [22–28]. Several implications follow from this general trend: first, the negative correlation between *α* and *β*-diversity exists among a set of non-Western populations practicing largely traditional subsistence lifestyles, demonstrating that the correlation is not entirely a Western versus non-Western phenomenon; second, the correlation is not associated with the particular subsistence lifestyle; and third, the correlation is more significant for unweighted *β*-diversity than for abundance weighted *β*-diversity. These three points, and the fact that the correlation is negative, are consistent with a neutral, diffusion-limited process accounting for most phylogenetic differences in gut microbiome communities between the African populations in our study. This does not argue against selection acting on specific bacteria according to their niche role, only that selection on broad subsistence type does not appear to determine the overall phylogenetic distance between populations [63].

The contribution of host genetics to gut microbiome composition remains an open question, with studies finding evidence for heritability of relative bacterial abundances or specific taxa [15, 63–68], and alternately, estimating that host genetics explain only a minor percentage of microbiome variation [69]. We do not know whether the observed correlations between bacterial composition and host genetic similarity that we find in the within-population analysis of the Hadza and Maasai, or in the joint analysis of geographic and relatedness across all population, is tracking differences in specific genetic factors that mediate interactions with commensal microbiota (e.g., inflammation response or mucin production genes), or the tendency for closely related individuals to live and/or work in the same places and hence have a greater degree of shared environment compared to unrelated individuals. The bacterial compositional differences seen between countries, between populations within a country, and the significant dependence on the geographic distance between individuals in a linear model, underscore the importance of physical separation on the distribution of gut bacteria among population groups. Longitudinal studies may be may be required to understand whether these correlations are plausibly due to bacterial dynamics within a population, while much larger cohorts are required for adequately powered statistical tests of whether these correlations are due to heritable host genetic factors.

The functional differences of the predicted metagenomic content of the gut microbiomes supports the hypothesis that there are both country-level and population-level differences in the distribution of functional pathways among the gut bacteria. We find that most imputed KEGG pathways that are more enriched in the USA compared to the two African countries as a whole are also more enriched in Botswana than in Tanzania. KEGG pathways with this enrichment pattern include categories that relate to the degradation of industrial compounds and by-products, such as bisphenol, xylene, DDT, and styrene. This pattern possibly reflects selection for increasing the abundance of bacteria that can degrade or metabolize environmental xenobiotic compounds.

The imputed bisphenol degradation pathway also has highest frequency in the USA, followed by Botswana, then Tanzania. Bisphenol is a common industrial organic compound used in many plastics and epoxies. The sampled African populations live far from industrial centers and arguably have less contact with plastics and industrial by-products compared to the US individuals; consequently, the frequency pattern of the bisphenol degradation pathway could indicate that the presences of bisphenol is influencing the US gut microbiome. A similar argument applies to the higher frequencies of imputed styrene degradation and xylene degradation pathways in the USA compared to Botswana and Tanzania.

Within Africa, we find that Botswana has a higher frequency of these industrial compound degradation pathways compared to Tanzania, including imputed DDT degradation pathways. Interestingly, Botswana, but not Tanzania, is one of nine countries worldwide that uses indoor residence spraying of traditional structures for control of malaria-carrying mosquitos [70, 71]. These results suggest potential metagenomic adaptation to increased exposure to industrial compounds in western populations, and to DDT in Botswanans.

There are caveats to interpretation of PICRUSt results; we do not know with certainty what the sources are that may explain these differences in imputed functional enrichment. Additionally, imputed gene content from reference strains may not adequately capture the gene content in strains that have diverged due to, for example, horizontal gene transfer and selection (e.g., antibacterial resistance). Shotgun sequencing of the gut bacteria will be required to directly verify the metagenomic functional differences observed here and to investigate potentially novel bacterial strains found in these Africa populations. The US population sampled here is the only population from an urban city in our study, which we may reasonably expect to contain more industrial pollutants in the general environment than in the environment of any of the populations we sampled in Africa. Consequently, it would be of interest to sample populations from Botswana and Tanzania that reside in major urban centers where there is more exposure to industrial pollutants, to see if their gut bacteria are enriched for functions more similar to what we see in the US population with regard to industrial by-product degradation and xenobiotic metabolism.

## Conclusions

The genetic and cultural diversity of Africans extends to the taxonomic diversity of their gut microbiomes. The gut bacteria in Botswana are relatively more similar to the USA, and a subset of traditional farmers has gut bacteria nearly indistinguishable from that in the US cohort. Correspondingly, the phylogenetic diversity between rural African populations can be as large as the differences we find between traditional and urban populations. In general, the regional phylogenetic distinction between Botswana and Tanzania exceed the distinction found between subsistence lifestyles. The factors causing a shift towards western microbiome compositions remain unknown but appear to have a regional component that is not entirely due to differences in agricultural, pastoral, or hunting-gathering subsistence modes.

## Methods

### Sampled populations

Ethnic groups, language, sample sizes, subsistence classifications, and sampling coordinates of populations are listed in Additional file 2: Table S1A. Written informed consent was obtained from all participants, and ethics/research approval and permits were obtained from the following institutions prior to the start of sample collection: NIMR, COSTECH, and Muhimbili University of Health and Allied Sciences in Dar es Salaam, Tanzania; The University of Botswana and the Ministry of Health in Gaborone, Botswana; IRB approval from the University of Pennsylvania. Samples were collected from Botswana during the wet season and the start of the dry season (January–April) and from Tanzania at the end of the dry season/start of the wet season (October–March). We recruited 114 adult participants (26 Bantu, 8 Herero, 20 San, 25 Hadza, 12 Sandawe, 12 Maasai, 11 Burunge) that practiced diverse modes of subsistence (such as pastoralism, agropastoralism, hunting and gathering, and mixed hunting and gathering). Demographic information including age, sex, ethnicity, and ancestry was recorded (for further participant details see Additional file 2: Table S1A). We provide a dietary literature review of our sampled populations in Additional file 2: Table S1W. Basic demographic data and fecal 16S rRNA V1-V2 sequences for healthy Philadelphians (US cohort) were collected from prior studies at the University of Pennsylvania [12, 32, 33]. All fecal and blood samples were extracted, sequenced, and analyzed using the same laboratory and computational pipelines, thereby reducing the impact of batch effects in the cross-population comparisons.

In Tanzania, samples were obtained from the Hadza hunter-gatherers who live in the Arusha and Shinyanga regions surrounding Lake Eyasi, the Maasai pastoralists from the northern Ngorongoro district, and the Burunge agropastoralists and Sandawe former hunter-gatherers who reside near each other in the Kondoa district in Central Tanzania (Fig. 1). Each of these four ethnic groups has a distinct dietary pattern. Hadza hunter-gatherers rely on local, natural resources that are structured by annual and seasonal changes in rainfall [31]. Specifically, Hadza diets are dominated by tubers, legumes, berries, baobab fruit, honey, and foraged plant material [57, 72]. The Sandawe are a former hunter-gatherer group that settled in villages and began farming in the nineteenth century. They primarily subsist on grains, with supplements of tubers and plant material gathered from the bush. Up until the mid-1800s, the Sandawe were a semi-nomadic hunter-gatherer population living in the savannahs of Tanzania. The Sandawe have admixed with neighboring populations of diverse ancestries who migrated into Tanzania within the past 5000 years [73]. The Sandawe also adopted the agropastoral subsistence practices of neighboring Bantu-speaking Turu, which comprises the bulk of their caloric intake, though they continue to supplement a small portion of their diet with hunting and gathering [54, 74].The Maasai are nomadic cattle, sheep, and goat herders living in the Ngorongoro highlands region. Maasai diets primarily consist of meat, milk, and blood, which are lactose rich and high in fat and cholesterol, though they supplement that diet with maize traded from neighboring groups [75]. The Burunge are settled farmers that also keep livestock, with a diet heavily dependent on millet and subsidized by cattle derived dairy and meat.

In Botswana, samples were obtained from western/northwestern regions from San populations who traditionally have practiced hunting and gathering (Naro, Kaukau, Ju|‘hoan!, Xoo) and from several agropastoralist populations (Kgalagadi, Tswana, Mophadima) that are classified here as “Bantu” based on their shared language family and broad subsistence practice, and one population, the Herero, who practice a pastoralist lifestyle. The traditional diet of San hunter-gatherers is composed of foraged meat, vegetables, fruits, and nuts, the latter of which contributed the largest percentage of dietary protein and calories [76–80]. Some San settlements receive a substantial component of their food from government sources [34, 35]. Bantu agropastoralists have diets mainly composed of sorghum, maize, millet, legumes, cucurbits (squash and melons), eggs, and seasonally available fruits in addition to goat, chicken, fish, and cattle meat [81]. Herero pastoralists have diets based on beef, milk, and milk products with supplements of goats, chickens, garden produce, foraged plants and animals, and bulk grains (especially ground corn) [82].

### Sample collection and storage

Participants produced a fecal sample in a sterile container that was immediately returned to researchers at the field site. A midsection sample of stool was harvested in a 5 ml container and immediately frozen in liquid nitrogen. Samples were later aliquoted into smaller 1.5 ml containers on dry ice in a fume hood to maximize storage space. The samples were stored at − 80 °C before transportation to the USA in dry ice, where it was again stored at − 80 °C until extraction.

### Biological sample processing and quantification

#### 16S rRNA gene sequencing and processing for microbiome sequencing

Total DNA from fecal materials was extracted using a PSP Spin Stool DNA Plus Kit (Stratec Molecular) with a modified bead-beating method [83]. PCR and extraction blanks were used to control for reagent and environmental contamination, and all extractions were conducted in a laminar flow hood, with equipment and consumables given UV irradiation for a minimum of 30 min prior to use. Eluted DNA was quantified by fluorometry and stored at − 20 °C. PCR reactions were performed in quadruplicate using the Accuprime system (Invitrogen) and barcoded composite primers with Illumina adapters to amplify the V1-V2 sections of the 16S rRNA genome (see Additional file 2: Table S1Y for 16S rRNA gene sequencing metadata and PCR conditions). The resulting 300–320 bp products were pooled and visualized by gel electrophoresis, followed by product purification using 1:1 volume of Agencourt AmPure XP beads (Beckman-Colter). Purified PCR products, including extraction and PCR blanks, had their final concentration determined with Qubit PicoGreen dsDNA BR assays (Invitrogen) and were pooled in equal amounts prior to Illumina Nextera XT library preparation (processed by the manufacturer’s protocol). Libraries were multiplexed on the Illumina MiSeq system and sequenced using 2 × 250 bp cycles. Sequence data are deposited under project accession PRJNA395034 in the NCBI Sequence Read Archive; sample details and individual accession numbers are included in the Additional file 2: Table S1A.

#### 16S rRNA processing and qPCR

In a separate extraction, total DNA from fecal materials was extracted from samples using a MO BIO PowerSoil DNA Isolation Kit (MO BIO Laboratories, Carlsbad, CA). Eight samples from each population were included save for the Herero, where only three were available, and the Bantu, where nine were available. No stool sample was available for the US individuals, and they were not included in this analysis. Each fecal sample was individually weighed, with samples ranging from 0.012 to 0.196 g. The samples were then processed according to manufacturer’s protocols, and eluted DNA was quantified by fluorometry and stored at − 20 °C. Bacterial abundance was quantified by qPCR amplification of the V1-V2 region of the 16S rRNA gene, with reactions performed in triplicate (25 μL each), using 1:1000 dilutions of DNA template. For qPCR, equal volumes of purified DNA of all samples were used in this assay. Primer and probe sequences are as follows: BSF8 (Forward) qPCR primer—5′-AGAGTTTGATCCTGGCTCAG-3′, BSR65/17 (Reverse) qPCR primer—5′-TCGACTTGCATGTRTTA-3′, Fluorescent dye (5′6 -FAM (Fluorescein)), landing sequence, dark quencher (3’ Black Hole Quencher®-1) 5′– /56-FAM/TAA + CA + C ATG + CA + A GT + C GA/3BHQ_1/ - 3′. *A + indicates a locked nucleic acid base. Primers and probes were purchased through Integrated DNA Technologies (IDT).

Prior research has indicated that the differences between 16S qPCR copy numbers produced from the same samples but extracted with both PSP and MoBio kit were statistically negligible [84]; thus, the MoBio extracts can serve as an accurate proxy for PSP extracts for 16S qPCR. The Bantu had a mean 16S rRNA gene copy number per gram of stool of 1.51 × 10^9^ ± 3.73 × 10^8^ SEM (standard error of the mean), the Burunge had 8.06 × 10^9^ ± 5.01 × 10^9^ SEM, the Hadza had 1.81 × 10^9^ ± 4.25 × 10^8^ SEM, the Herero had 1.68 × 10^9^ ±  4.73 × 10^8^ SEM, the San had 1.41 × 10^9^ ±  3.27 × 10^8^ SEM, the Maasai had 1.81 × 10^9^ ±  3.35 × 10^8^ SEM, and the Sandawe had 1.87 × 10^9^ ±  2.12 × 10^8^ SEM .

#### OTU clustering

Bacterial 16S rRNA reads were analyzed using the Quantitative Insights into Microbial Ecology (QIIME) software package [85]. During the quality-filtering process, reads were removed from the analysis if they did not match Golay error-corrected barcode with less than two mismatches, if the read pairs could not be joined with an overlapping sequence of less than 35 bp, if they had a homopolymer sequence (repeated base call) greater than 6 bp, and if they had more than two ambiguous base calls (N’s). Operational taxonomic units (OTUs) were created by single-linkage clustering the reads using Swarm [86] and removing OTUs comprised of only a single or pair of reads. Representative sequences from each OTU were aligned using the PyNAST aligner [87], and a phylogenetic tree was inferred using FastTree v. 2.1.3 [88] after applying the standard Lane mask for 16S sequences. [89] As an additional quality control step, all OTUs were tested for correlations between the proportional abundance of the OTU and the post-PCR amplicon concentration of a sample using the method developed by Jervis-Brady et al. [90] as implemented in the contam_test program for R (https://github.com/eclarke/eclectic). A negative correlation indicates a potential contaminant: an increasing proportional abundance of that OTU in correlation with lower sample biomass (as implied by lower amplicon concentration) suggests that the increased proportional abundance of that OTU comes in as part of the reagents, and is not truly part of the sample. Correlation significance is assessed using Pearson’s rho, and OTUs with a significant negative correlation are considered contaminants and removed. Final OTU sequences are listed in Additional file 2: Table S1Z. Taxonomic assignments were generated using the Greengenes 16S database v. 13_8 [91] (Additional file 2: Table S1AA) and OTUs mapping to chloroplast or mitochondrial sequences were removed. All OTUs are denoted by the prefix “denovo” since they are determined without use of reference sequences. OTU and MRT abundances are measured as the proportion of the total reads per individual.

#### Host genotyping and genetic relatedness

DNA was extracted from white blood cells using a salting out method (Gentra Puregene) and 97 of the 114 African individuals were genotyped on the Illumina Omni5M Exome array that includes a small number of indels and ~ 4.5 million SNPs (see Additional file 2: Table S1A). In collaboration with the Cancer Genomics Research laboratory (CGR) at NIH, array intensity data was clustered and all genotypes were called based on standard operating procedures using the hg19/37 SNP coordinates in the Illumina software *GenomeStudio*. See Crawford et al. 2017 [92] for further details on this genotype callset. We retained the segregating autosomal biallelic single nucleotide polymorphisms (SNPs) over the 97 individuals, and variants were pruned to be in approximate linkage equilibrium, *r*^2^_LD_ < 0.1, using plink [93](plink --indep-pairwise 200 kb 20 0.1), leaving 158,891 SNPs for genetic relatedness estimation. From these sites, we constructed (A) the estimated pairwise identity-by-descent fraction among all pairs of individuals from the same population (plink --genome, see Additional file 3), and (B) a genetic relationship matrix between all pairs of individuals i and j from the standardized genotype vectors using the *Genome-wide Complex Trait Analysis* (GCTA) software [94] (--make-grm-gz) for subsequent analyses (see Additional files 4 and 5).

#### qPCR for *Entamoeba histolytica*

Primers, probes, and protocols for the qPCR, including methods for generating a recombinant plasmid containing target *E. histolytica* sequence to make a standard curve, were taken from Mejia et al. (2013) [95]. qPCRs were run on a QuantStudio7 Flex Real-Time PCR system.

### Statistical methods

#### Diversity and richness measurements

Diversity metrics (α and *β*-diversity) were quantified using all 17,861 taxonomically mapped OTUs using QIIME [96, 97]. QIIME was also used to calculate UniFrac distances, which are an estimate of the fraction of the total branch length over the bacterial phylogenetic tree that is not shared by two bacterial communities [96, 97]. Unweighted UniFrac distance is based on the presence/absence of bacteria [96], while weighted UniFrac distance weights the shared branches in the phylogenetic tree by abundance [97]. Species accumulation curves were calculated using the *specaccum* function from the vegan library for R.

#### Phylogenetic variation among groups

PERMANOVA tests between groups were computed with Python package *scikit-bio* (scikit-bio.org), using 50,000 permutations. The PERMANOVA test statistic is the ratio of between-group to within-group variance, and is sensitive to whether the mean separation between groups is larger than the mean variance within groups.

#### Principal coordinate analysis

Principle coordinate analysis (PCoA) was computed using the Python package *scikit-bio 0.5.1* (scikit-bio.org).

#### Analysis of differentially abundant taxa

We used the Analysis of Composition of Microbiomes (ANCOM) method to detect differentially abundant taxa between groups [40], as implemented in the Python *scikit-bio* 0.5.1 package. The ANCOM method accounts for the simplex nature of compositional data, and so does not suffer from spurious negative correlations imposed by the fact that (relative) abundances across all bacteria must sum to one within a given bacterial community. This method tests for taxa that vary significantly among groups more than a significant number of the other taxa. Consequently, if a large number of taxa all vary similarly among the groups, then none of these will show up as significantly varying compared to the other taxa. As such, this is a sensitive test for taxa that vary significantly and in an unusual way compared to the other taxa. For all tests, we used the default “one way ANOVA” base test, with a significance threshold of 0.05, tau parameter 0.99, and theta parameter 0.25, and we used the Holm-Bonferroni multiple testing correction.

For these analyses, we used abundances per mapped genus that have at least 0.1% mean abundance in at least one of the eight populations for the between continent comparison, and at least 0.1% mean abundance in at least one of the seven African populations for Africa-only comparisons. Since we are testing for difference between groups for each taxa, we rescaled the relative abundance by a constant factor and re-centered the relative abundances by adding a constant for each taxa, such that the rescaled relative abundances span from ~1/*N* to 1, where *N* is the total number of samples. The ANCOM analysis tests for differences between groups using the logarithm of the rescaled abundances. Since the logarithm cannot handle zero values, the choice of ~1/*N* as the minimum rescaled value avoids this issue. For the between continent comparison, all individuals are used, *N* = 126. For the Africa-only comparison we used *N* = 114. The rescaled relative abundances are given by *X* = (*x*−*A*)/ (*B*−*A*), where *x* is the original relative abundance, *A* = Min (*x*)-1/*N*, and *B* = Max (*x*). Note that the rescaling is done separately when using all samples (*N* = 126) or the Africa-only samples (*N* = 114).

#### Functional metagenomic analysis

Subsampled reads were subjected to closed reference OTU picking against the Green Genes reference taxonomy (Greengenes database, May 2013 version; http://greengenes.lbl.gov) using the pick_closed_reference_otus.py script in QIIME [85] using 97% identity. Metagenomes from bacterial OTUs were imputed with PICRUSt on the online Galaxy interface (http://huttenhower.sph.harvard.edu/galaxy). For each individual and each KEGG pathway (*N*_KEGG_ = 328), PICRUSt calculates the cumulative gene count across all OTUs that overlap the pathway, which are then normalized into a pathway abundance. The data were analyzed statistically by using STAMP v. 2.0.6. In this data set, the highest pathway frequency values are on the order of 0.1–1%, while the smallest non-zero pathway frequencies are on the order of 10^−7^ to 10^−6^%. The Nearest Sequenced Taxon Index (NSTI), which measures the phylogenetic distance between observed OTU sequences and the reference, has a mean and standard deviation of 0.140 and 0.037 across all samples, with 10th and 90th percentiles 0.095 and 0.186 (Additional file 2: Table S1AB). For multiple (> 2) populations, ANOVA and Tukey-Kramer post-hoc tests were performed. Two-group comparisons were done with White’s non-parametric t-test with two-sided confidence intervals obtained by bootstrapping. Multiple tests were controlled with FDR correction calculated by the Benjamini-Hochberg method.

For a given pathway k, the relative abundance difference between two groups A and B, *R*_*k*_(*A*, *B*), is defined by ${R}_k\left(A,B\right)=\frac{X(A)-X(B)}{\left(X(A)+X(B)\right)/2}$. Across all pathways *k* we find a significant, positive, correlation between *R*_*k*_(*Africa*, *U*. *S*.) and *R*_*k*_(*Tanzania*, *Botswana*) (Spearman rank correlation 0.51, *p* value < 10^−22^) (Additional file 2: Table S1 AC).

PICRUSt relies on the assumption that the bacterial strains in each sample have the same gene content as database strains used for the analysis, which can be inaccurate when strains vary substantially in gene content. However, it does not appear that the above correlation in pathway abundances can be explained by annotation biases alone. For example, if OTUs from Tanzania had a lower mapping rate to known taxa compared to Botswana, then this would results in lower abundances across all pathways in Tanzania compared to Botswana; this is not what we observe, as many pathways have a higher abundance in Tanzania than Botswana. The above correlation could only be explained by OTU annotation biases that (A) happen to impact certain pathways more than others and (B) have the same mapping biases between Tanzania and Botswana as between the USA and Africa. While we cannot rule this out, it would require several biases to align in direction.

#### BMI correlation

All of the individuals in our study have BMI measurements, which allowed testing for correlations between *α*-diversity and BMI. The BMI values were regressed on age and sex, and the residuals were tested for correlation with the Shannon diversity index. The “population re-centered” BMI residuals are computed as follows: the mean BMI residual is computed for each population, and for every individual in this population this value gets subtracted from their BMI residual.

#### Linear regression

Linear modeling and least squares fitting of UniFrac distances as a function of host geographic separation and host genetic relatedness were computed using the Ordinary Least Squares routines in the python package *statsmodels*.

## Additional files

Additional file 1: All supplementary figures are listed in Additional_File_1.pdf. (PDF 4060 kb)

Additional file 2: All supplementary tables are listed in Additional_File_2.xls. (XLSX 8380 kb)

Additional file 3: The estimated identity-by-descent used for the within-population correlation between UniFrac distance and host relatedness, as calculated using the plink --genome routine on the Illumina Omni 5 M genotype array dataset for 97 African individuals. (IBD 104 kb)

Additional file 4: Genetic relationship matrix file set used for linear regression analysis of UniFrac dependence on host relatedness and host geographic separation. This is constructed using the GCTA software (--make-grm), based on Illumina Omni 5 M genotype array dataset for 97 African individuals, and contains the (anonymized) individual identification codes. (ID 1 kb)

Additional file 5: Contains the genetic similarity values, where each row corresponds to a pair of individuals. (GZ 47 kb)
