# Supplementary material for: Population structure of human gut bacteria in a diverse cohort from rural Tanzania and Botswana
Source: Genome Biol. 2019 Jan 22;20:16. doi: 10.1186/s13059-018-1616-9 (PMC6341659; doi:10.1186/s13059-018-1616-9)
Supplement: Supplementary file 1 — All supplementary figures are listed in Additional_File_1.pdf. (PDF 4060 kb) [file 13059_2018_1616_MOESM1_ESM.pdf]

Additional File 1: Fig. S1

Unassigned reads that were not classified into any known taxa within the kingdom Bacteria. A) The mean unassigned abundance per individual per population. B) The mean number of unassigned OTUs per individual per population. C) The mean number of unassigned reads per individual per population.

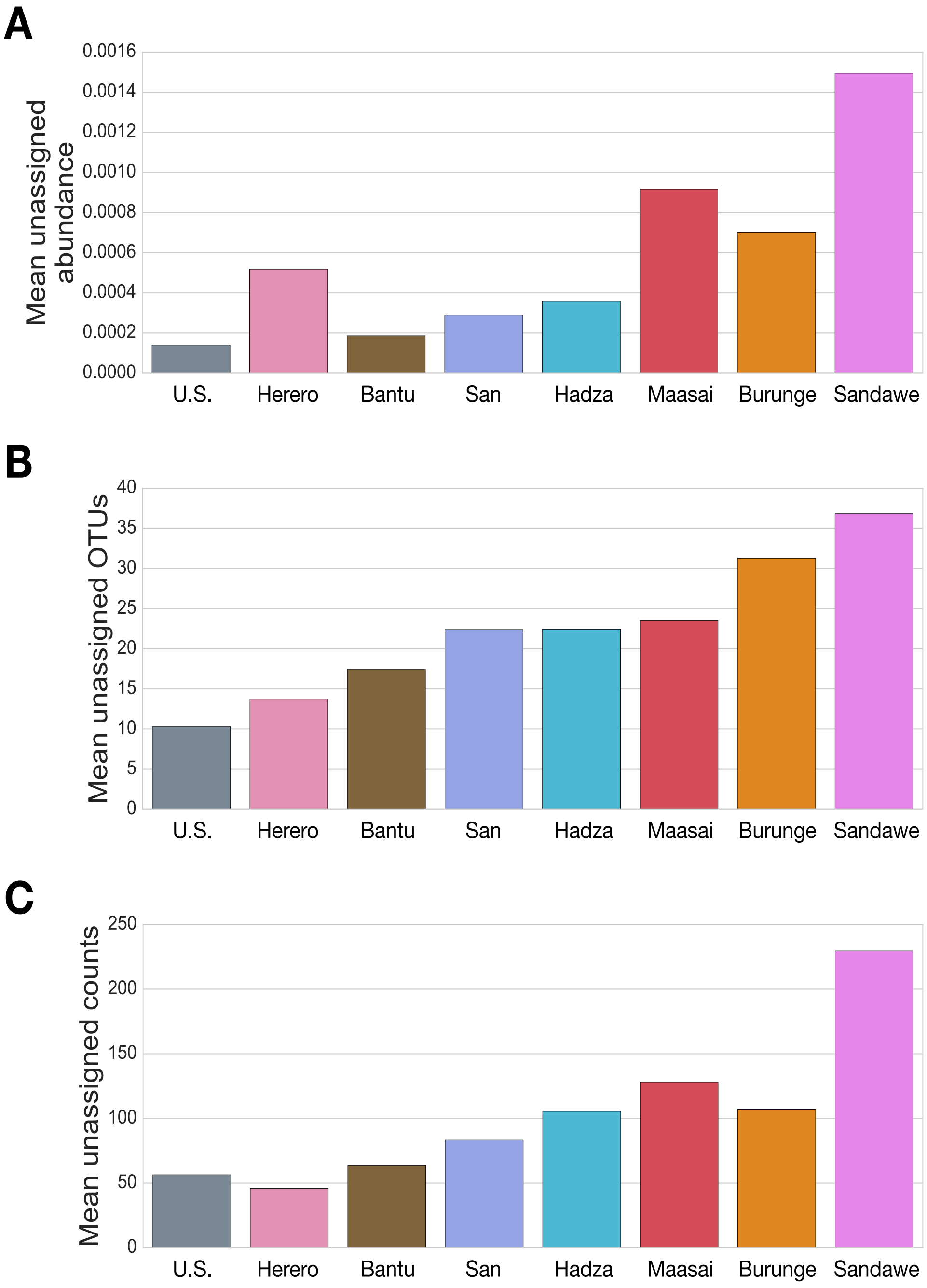

Additional File 1: Fig. S2

Population Averaged Species Accumulation Curves showing OTUs greater than 0.01% abundance for each population.

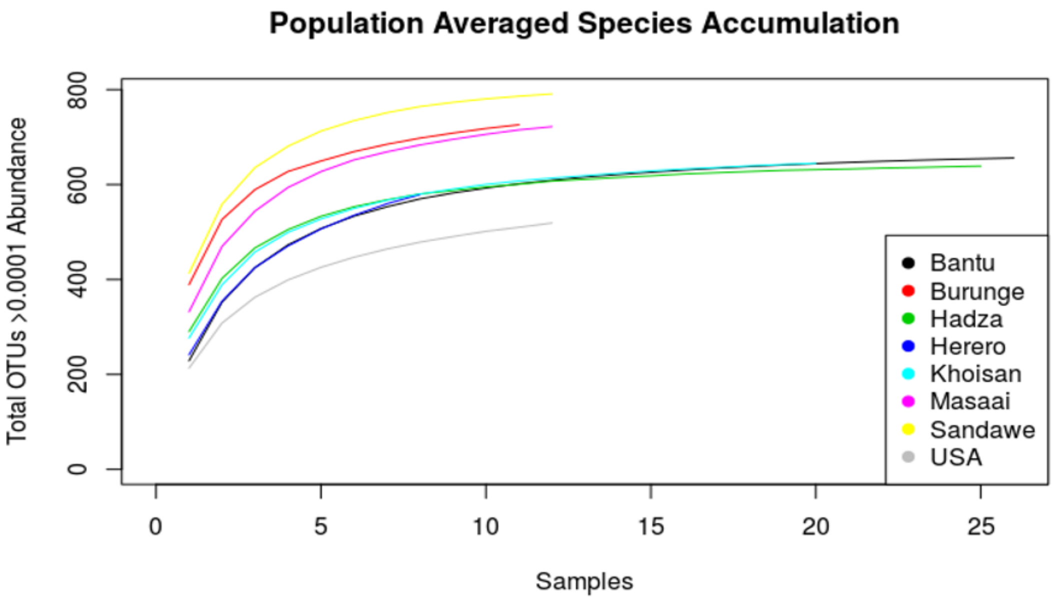

Additional File 1: Fig. S3

A) 16S rRNA Copy Number per uL of sample within each population. Lines through the center of the boxes show the median, and thetop and bottom are the 25th and 75th percentiles. Dashed line in center represents the group mean for all populations, against which a global ANOVA for all populations is derived (adjusted p=1e-06). B) 16S rRNA Copy Number per gram of stool within each population. Global ANOVA for all populations is not significant (adjusted p=0.27). Two samples within the Burunge had values high enough that they are plotted separately above the main boxplot (this was also done for all subsequent panels in S15). C) 16S rRNA Copy Number per gram of stool within each African subsistence group. Global ANOVA for all subsistence groups is not significant (adjusted p=0.58). D) 16S rRNA Copy Number per gram of stool within Botswana and Tanzania. Wilcoxon Rank -Sum test indicates that significant differences in copy number exist between the two countries (FDR corrected p=0.023). E) Linear regression on 16S rRNA Copy Number per gram of stool to Shannon Diversity per sample, colored by Population, and showing 95% confidence intervals.

A

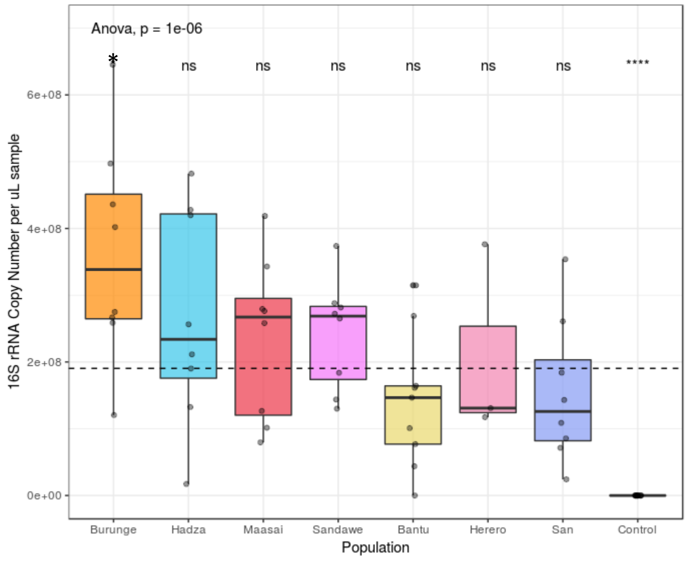

B

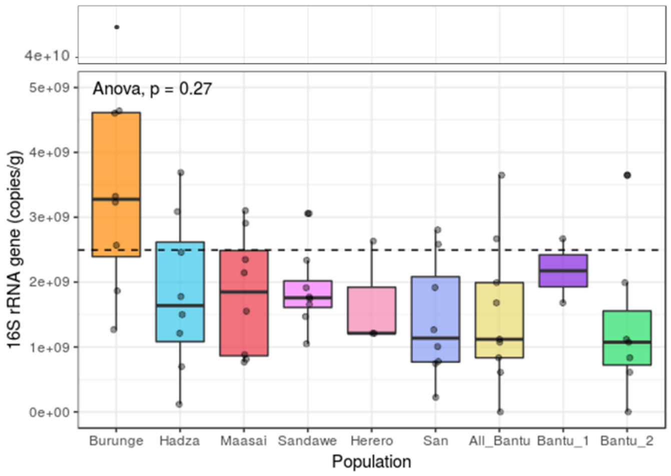

C

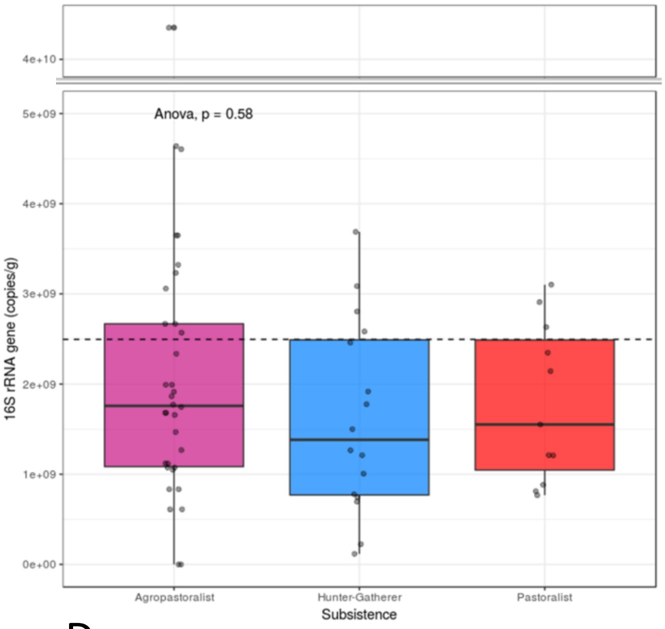

Anova, p = 0.58
16S rRNA gene (copies/g)
4e+10
5e+09
4e+09
3e+09
2e+09
1e+09
0e+00
Agropastoralist
Hunter-Gatherer
Pastoralist
Subsistence

* D

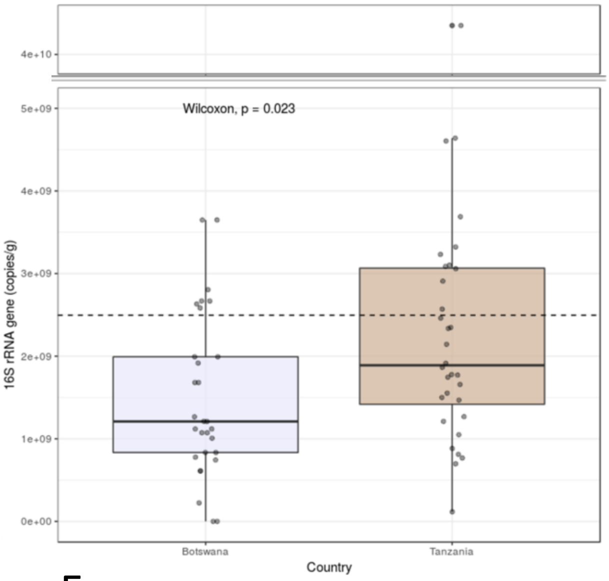

Wilcoxon, p = 0.023
16S rRNA gene (copies/g)
4e+10
5e+09
4e+09
3e+09
2e+09
1e+09
0e+00
Botswana
Tanzania
Country

E

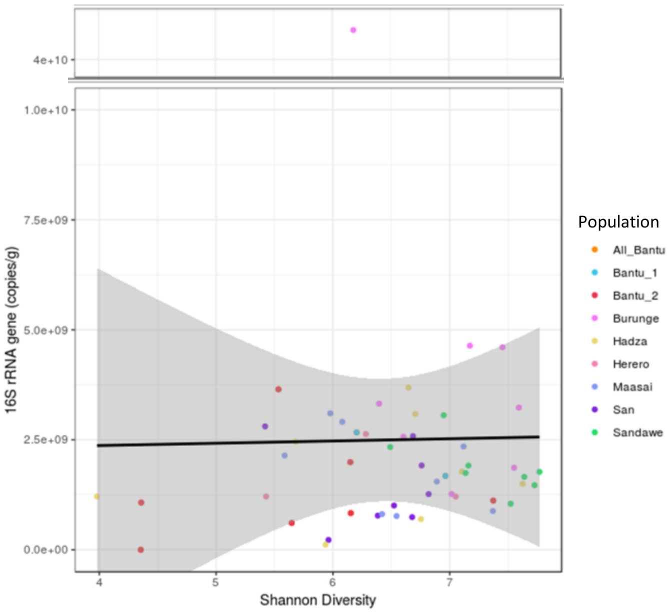

16S rRNA gene (copies/g)
4e+10
1.0e+10
7.5e+09
5.0e+09
2.5e+09
0.0e+00
4
5
6
7
Shannon Diversity
Population
All_Bantu
Bantu_1
Bantu_2
Burunge
Hadza
Herero
Maasai
San
Sandawe

Additional File 1: Fig. S4

Comparison of the Shannon Index between allreads and rarefied data (read counts subsampled down to 5,000 reads per individual), shown for all individuals.

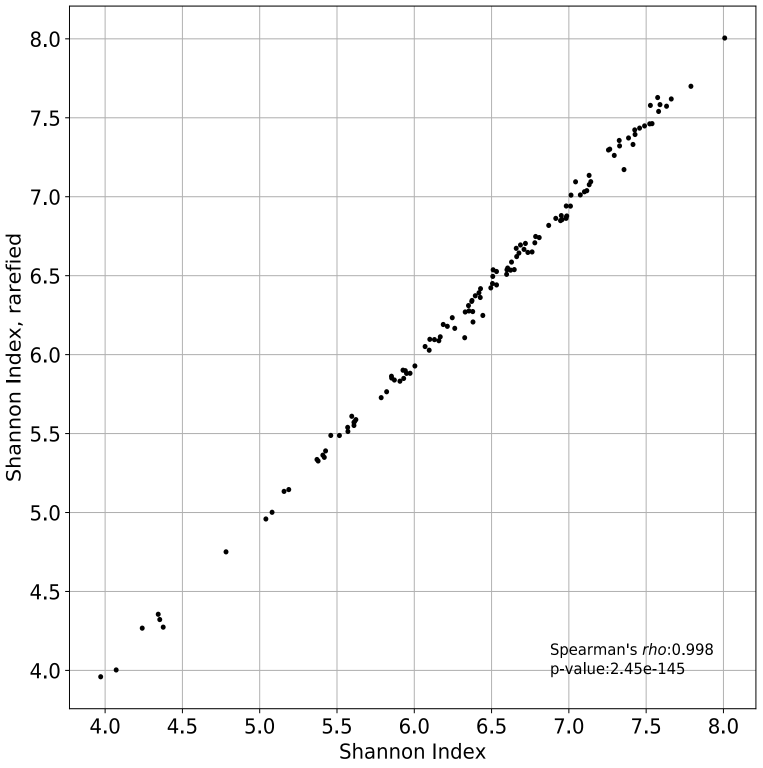

Additional File 1: Fig. S5

Similar to Fig.3 D, E where the pairs of individuals used for calculating the mean unweighted (A) and weighted (B) UniFrac distances are restricted to be individuals from the same sampling location as well as from the same population group.

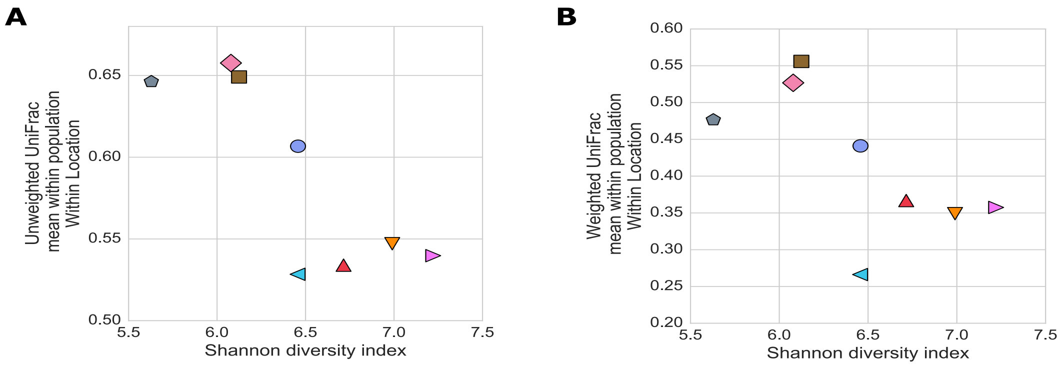

Additional File 1: Fig. S6

Similar to Fig. 3D,E, where the OTU data is rarefied to 5,000 counts per individual, Showing unweighted (A) and weighted (B) UniFrac distances.

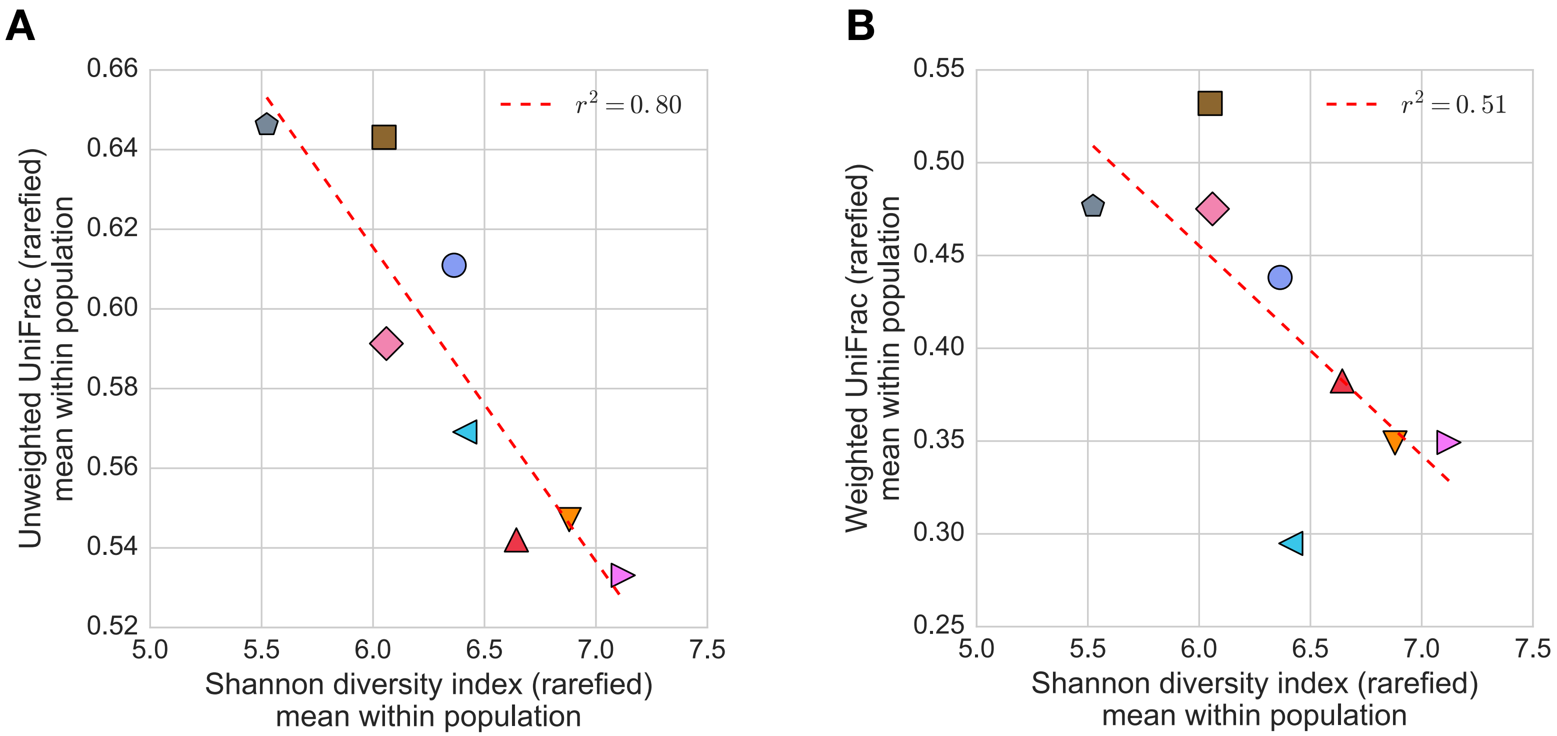

Additional File 1: Fig. S7

Comparison of the unweighted UniFrac distances when using all reads versus the rarefied data (read counts subsampled down to 5,000 reads per individual), shown for all pairs of individuals.

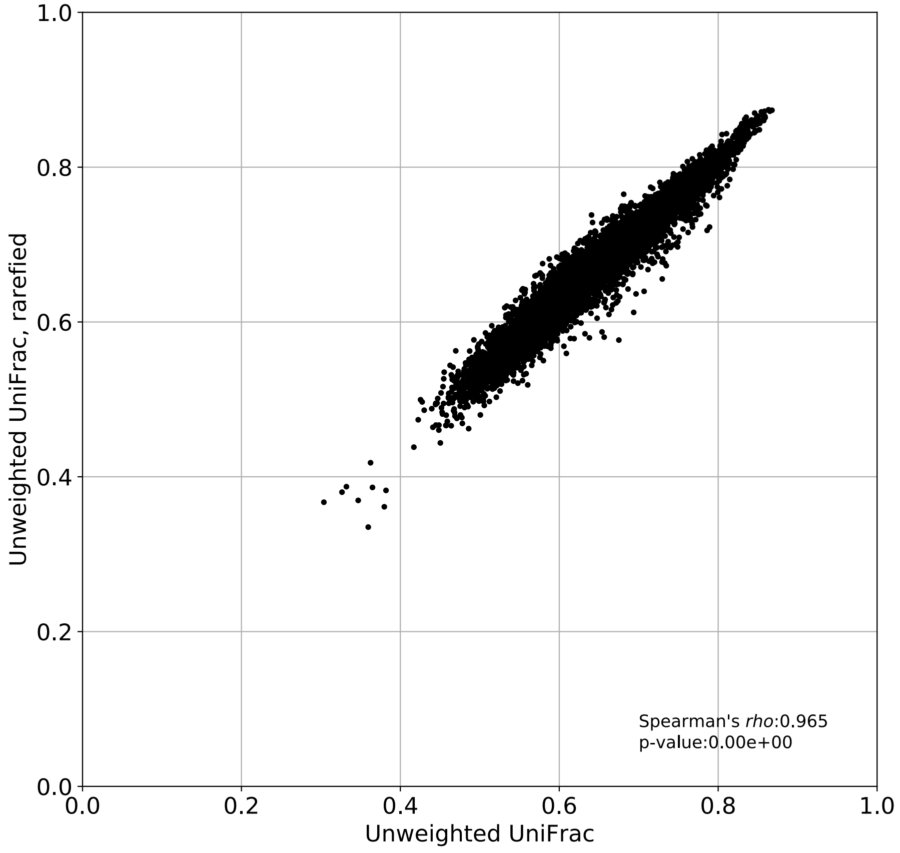

Additional File 1: Fig. S8

Comparison of the weighted UniFrac distances when using all reads versus the rarefied data (read counts subsampled down to 5,000 reads per individual), shown for all pairs of individuals.

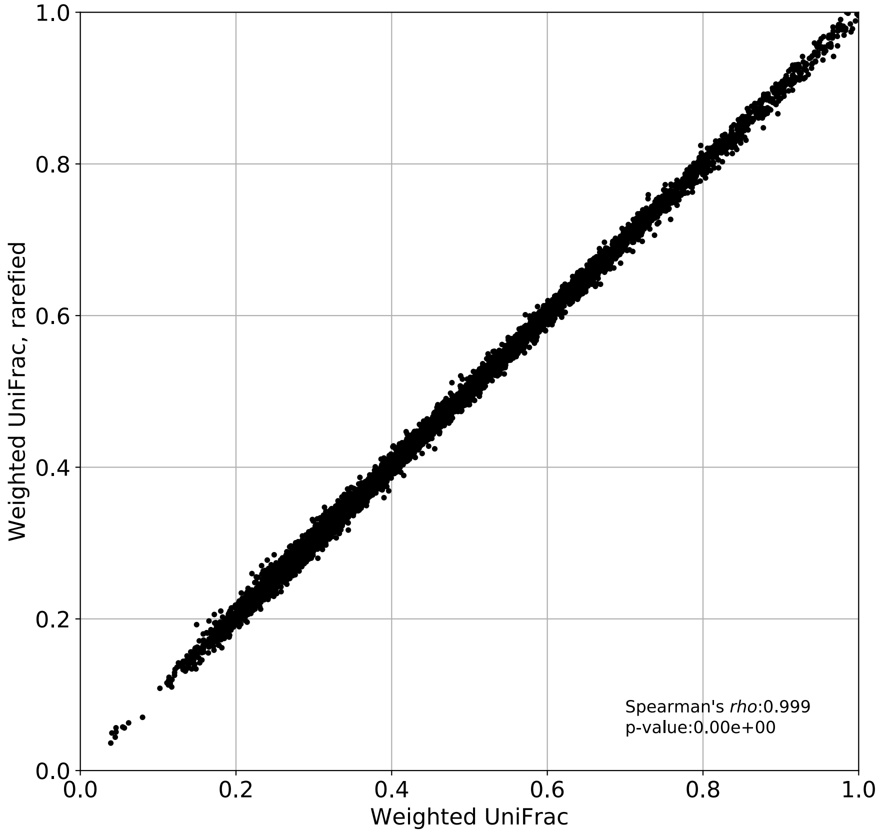

Additional File 1: Fig. S9

Similar to Fig. 3D,E, using the Bray-Curtis dissimilarity as the measure of beta-diversity between hosts.

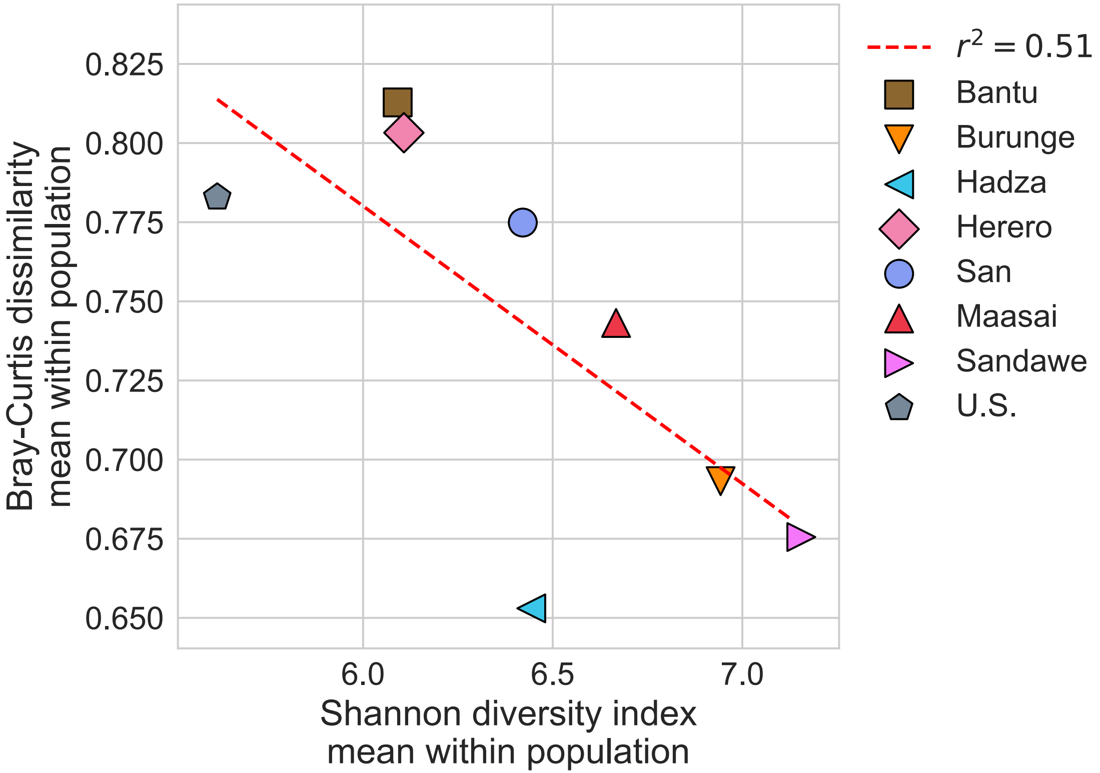

Additional File 1: Fig. S10

Correlation plot between the Bray-Curtis dissimilarity and (A) unweighted and (B) weighted UniFrac distances.

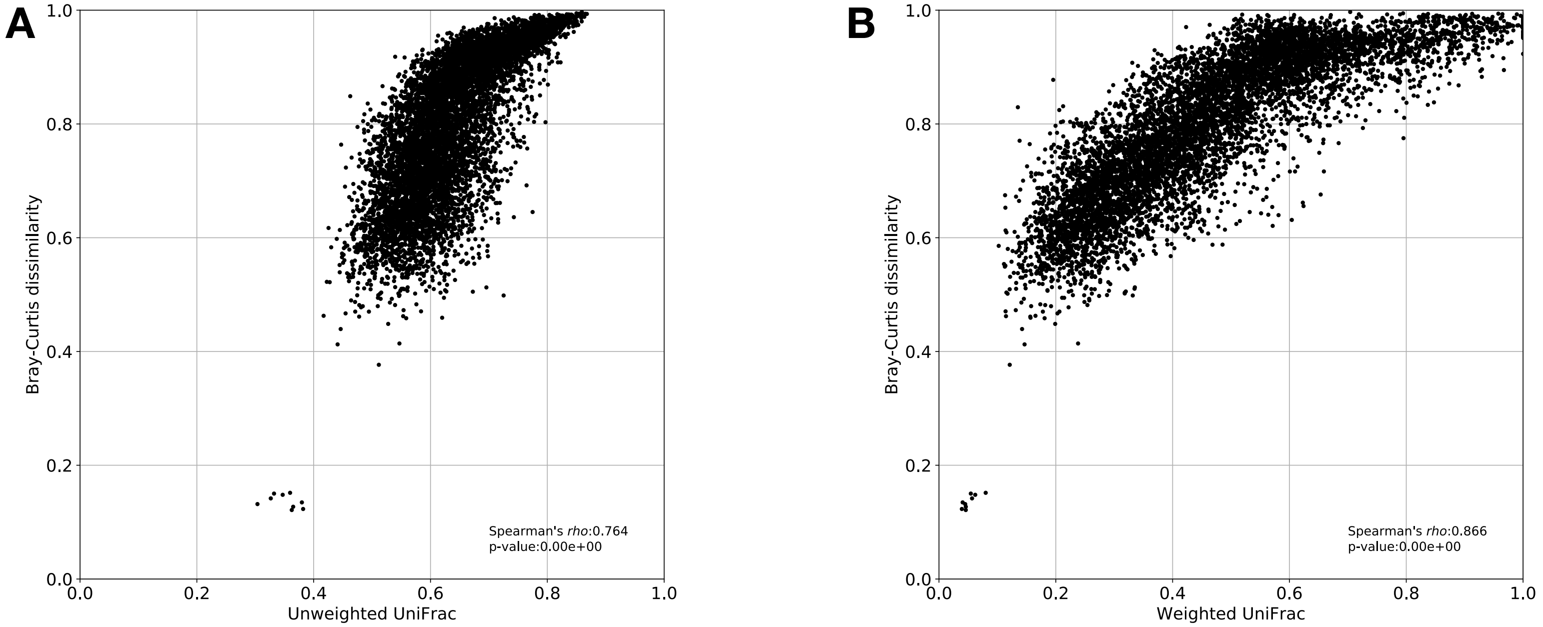

Additional File 1: Fig. S11

Mean pairwise compositional distance between all pairs of populations, for (A) unweighted and (B) weighted UniFrac distances.

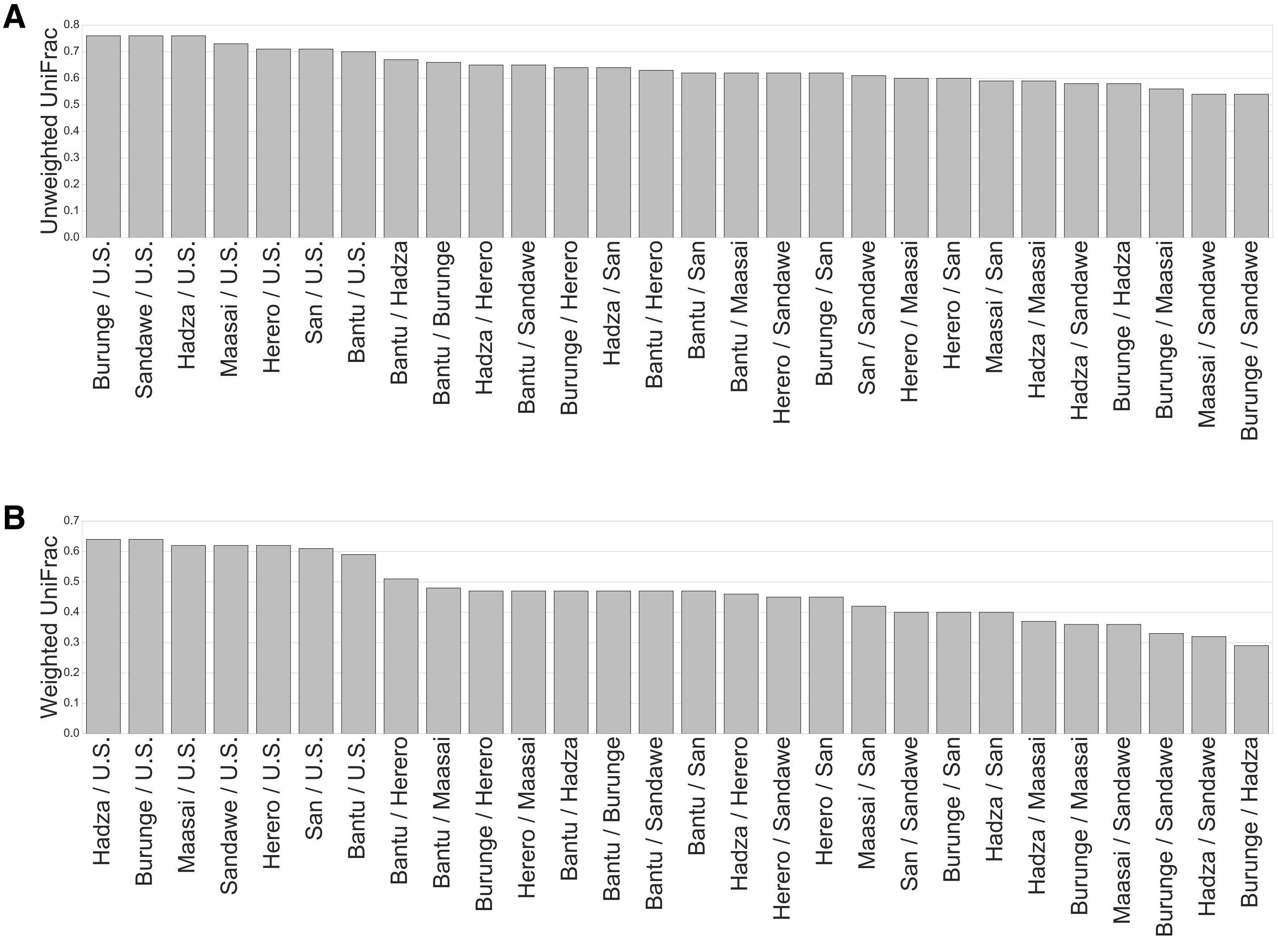

Additional File 1: Fig. S12

Principal Coordinate Analysis of unweighted UniFrac distances, analogous to Fig.5.

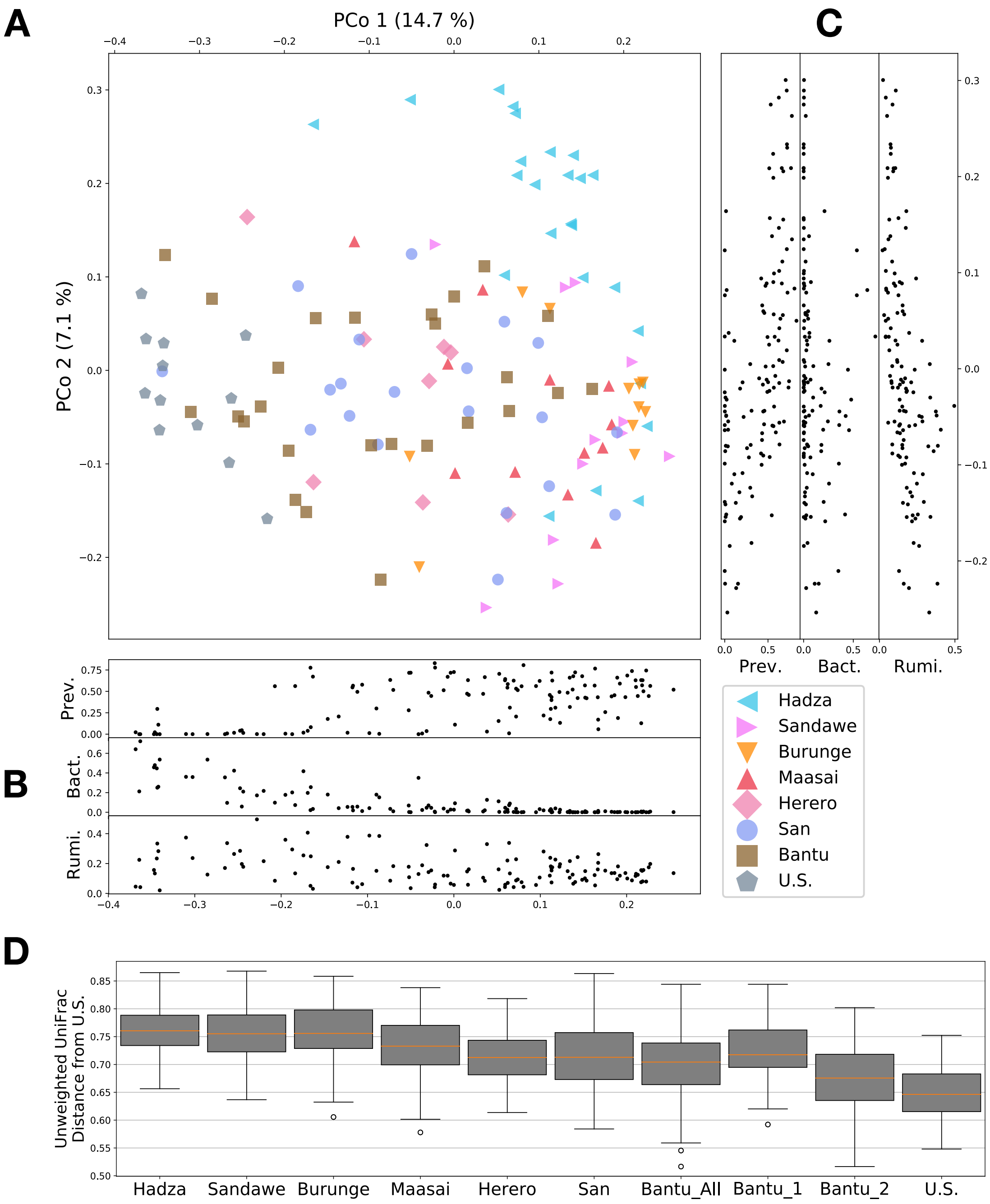

Additional File 1: Fig. S13

A) Relative pathway frequency differences between Tanzania and Botswana (x-axis) and Africa and the U.S. (y-axis) for all KEGG pathways analyzed (N=328). Marker size is proportional to the mean pathway frequency across the U.S. and Africa. Dotted lines demark relative differences of ± 15%. Pathways in red denote those shown in Additional File 2: Table S1 (absolute relative differences > 15% and FDR < 0.1). Panels B), C), and D) show the pathway frequency distribution boxplots per population for the DDT degradation, xylene degradation, and bisphenol degradation pathways, respectively. Lines through the center of each box show the median, the star represents the mean, and the top and bottom are the 25th and 75th percentiles.

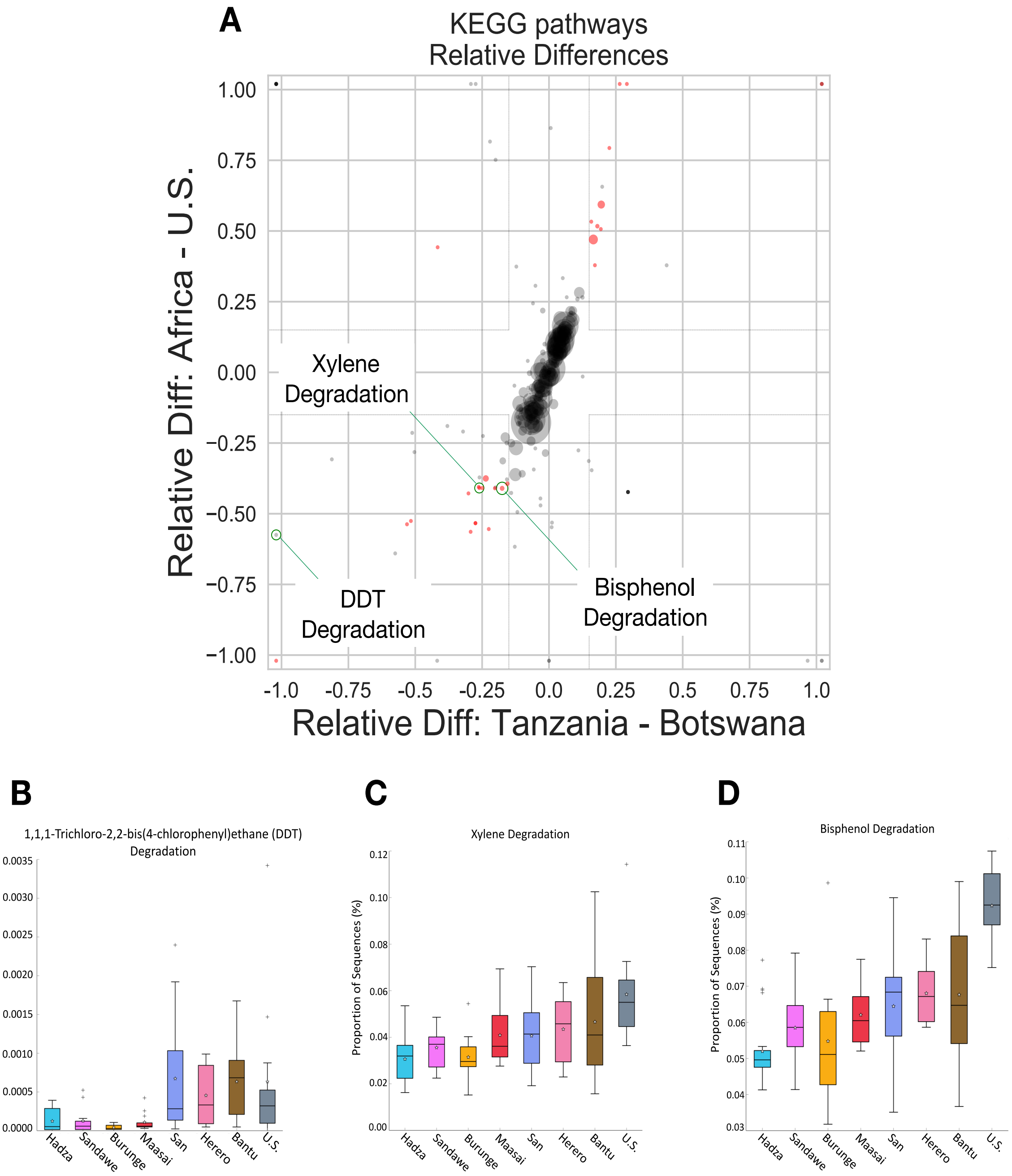

Additional File 1: Fig. S14

Comparison of reported relative abundances (mean +/-standard deviation) of our study cohort with for global populations, adapted from Smits et al. The taxa included are the bacterial Families *Bacteroidaceae*, *Prevotellaceae*, *Spirochaetes*, *Succinivibrionaceae*, and the bacterial Phylum *Verrucomicrobia* , shown on a linear abundance scale.

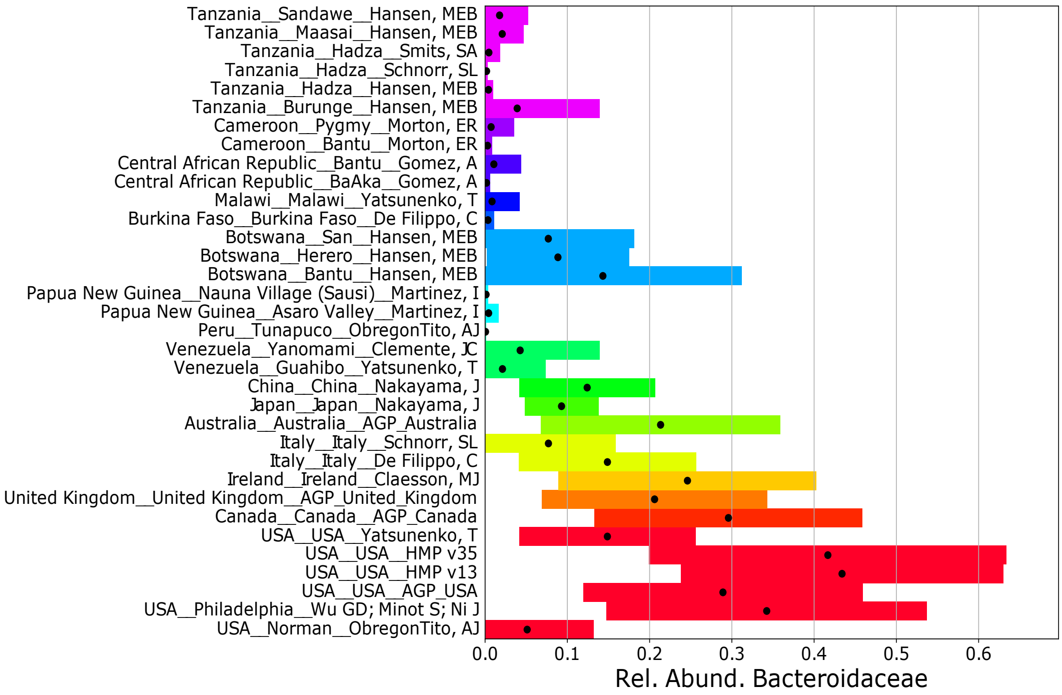

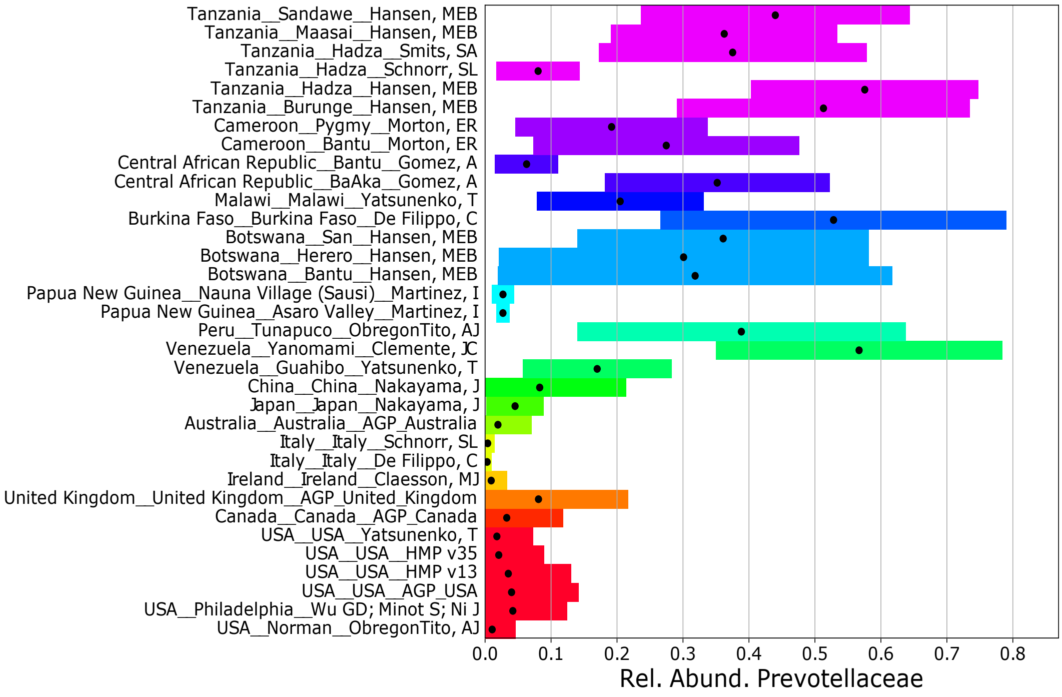

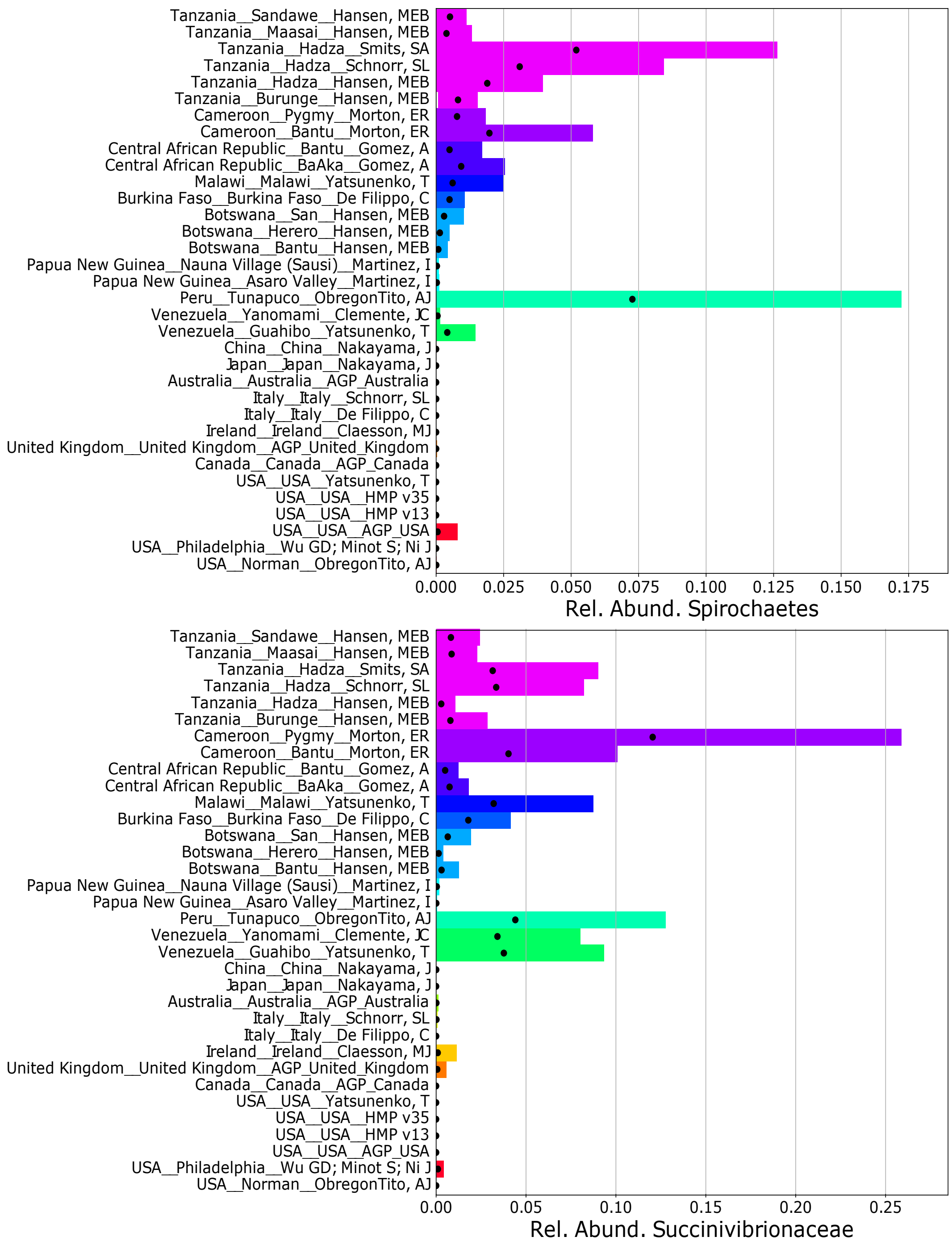
Tanzania__Sandawe__Hansen, MEB
Tanzania__Maasai__Hansen, MEB
Tanzania__Hadza__Smits, SA
Tanzania__Hadza__Schnorr, SL
Tanzania__Hadza__Hansen, MEB
Tanzania__Burunge__Hansen, MEB
Cameroon__Pygmy__Morton, ER
Cameroon__Bantu__Morton, ER
Central African Republic__Bantu__Gomez, A
Central African Republic__BaAka__Gomez, A
Malawi__Malawi__Yatsunenko, T
Burkina Faso__Burkina Faso__De Filippo, C
Botswana__San__Hansen, MEB
Botswana__Herero__Hansen, MEB
Botswana__Bantu__Hansen, MEB
Papua New Guinea__Nauna Village (Sausi)__Martinez, I
Papua New Guinea__Asaro Valley__Martinez, I
Peru__Tunapuco__ObregonTito, AJ
Venezuela__Yanomami__Clemente, JC
Venezuela__Guahibo__Yatsunenko, T
China__China__Nakayama, J
Japan__Japan__Nakayama, J
Australia__Australia__AGP_Australia
Italy__Italy__Schnorr, SL
Italy__Italy__De Filippo, C
Ireland__Ireland__Claesson, MJ
United Kingdom__United Kingdom__AGP_United_Kingdom
Canada__Canada__AGP_Canada
USA__USA__Yatsunenko, T
USA__USA__HMP v35
USA__USA__HMP v13
USA__USA__AGP_USA
USA__Philadelphia__Wu GD; Minot S; Ni J
USA__Norman__ObregonTito, AJ
0.000 0.025 0.050 0.075 0.100 0.125 0.150 0.175
Rel. Abund. Spirochaetes
0.00 0.05 0.10 0.15 0.20 0.25
Rel. Abund. Succinivibrionaceae

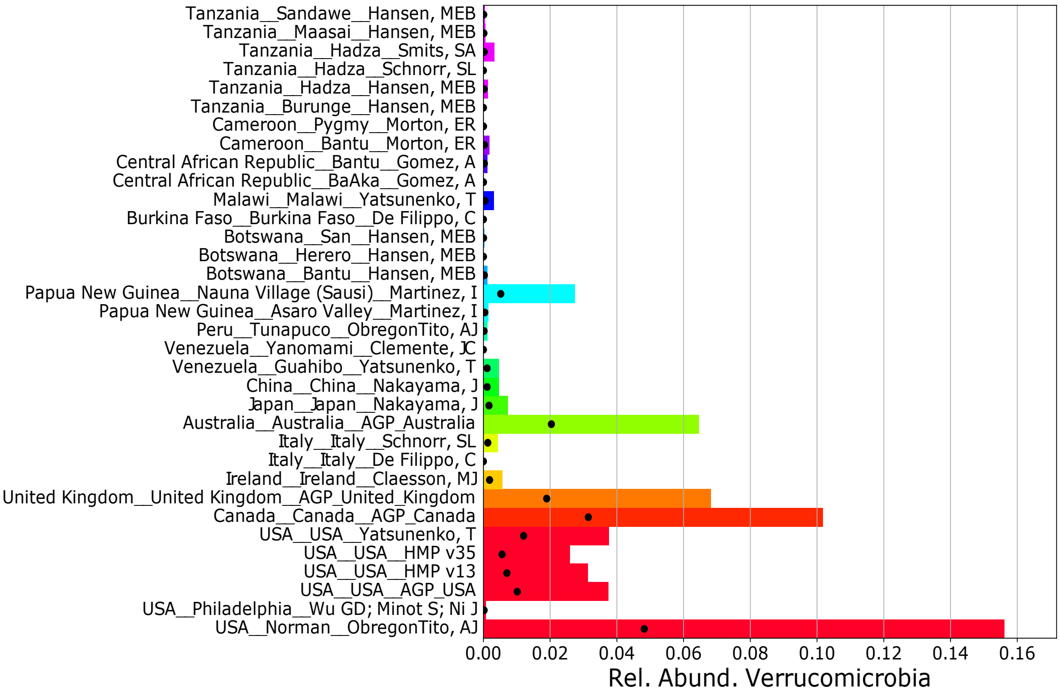

Tanzania__Sandawe__Hansen, MEB
Tanzania__Maasai__Hansen, MEB
Tanzania__Hadza__Smits, SA
Tanzania__Hadza__Schnorr, SL
Tanzania__Hadza__Hansen, MEB
Tanzania__Burunge__Hansen, MEB
Cameroon__Pygmy__Morton, ER
Cameroon__Bantu__Morton, ER
Central African Republic__Bantu__Gomez, A
Central African Republic__BaAka__Gomez, A
Malawi__Malawi__Yatsunenko, T
Burkina Faso__Burkina Faso__De Filippo, C
Botswana__San__Hansen, MEB
Botswana__Herero__Hansen, MEB
Botswana__Bantu__Hansen, MEB
Papua New Guinea__Nauna Village (Sausi)__Martinez, I
Papua New Guinea__Asaro Valley__Martinez, I
Peru__Tunapuco__ObregonTito, AJ
Venezuela__Yanomami__Clemente, JC
Venezuela__Guahibo__Yatsunenko, T
China__China__Nakayama, J
Japan__Japan__Nakayama, J
Australia__Australia__AGP_Australia
Italy__Italy__Schnorr, SL
Italy__Italy__De Filippo, C
Ireland__Ireland__Claesson, MJ
United Kingdom__United Kingdom__AGP_United_Kingdom
Canada__Canada__AGP_Canada
USA__USA__Yatsunenko, T
USA__USA__HMP v35
USA__USA__HMP v13
USA__USA__AGP_USA
USA__Philadelphia__Wu GD; Minot S; Ni J
USA__Norman__ObregonTito, AJ
0.00
0.02
0.04
0.06
0.08
0.10
0.12
0.14
0.16
Rel. Abund. Verrucomicrobia

Additional File 1: Fig. S15

Same as Additional File 1: Fig. S14 except shown on a log abundance scale.

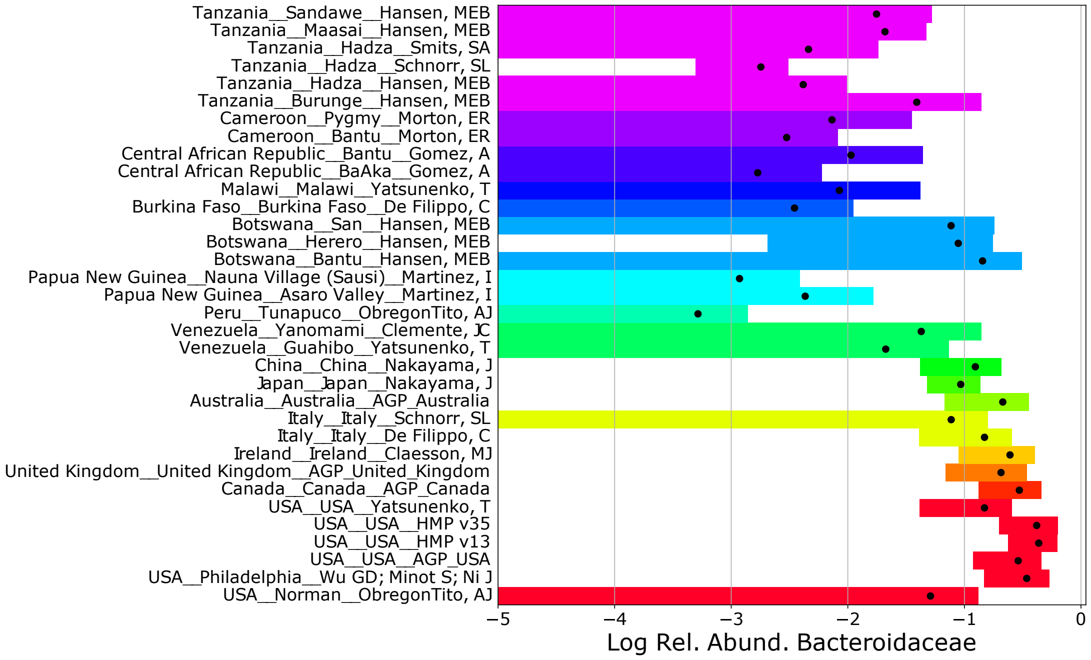

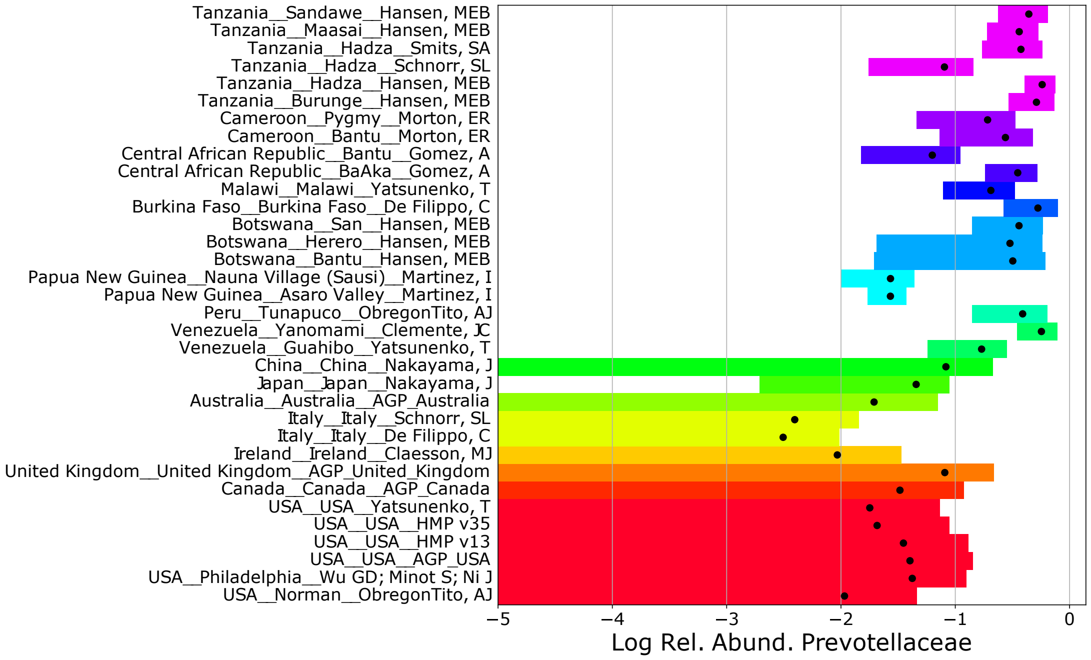

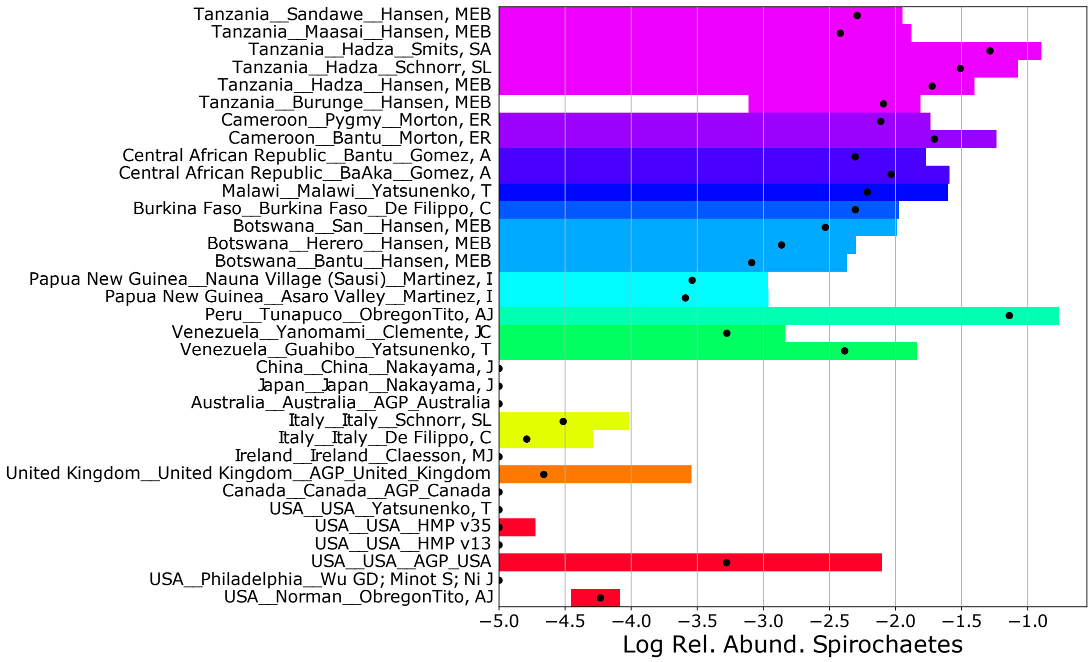

Tanzania__Sandawe__Hansen, MEB
Tanzania__Maasai__Hansen, MEB
Tanzania__Hadza__Smits, SA
Tanzania__Hadza__Schnorr, SL
Tanzania__Hadza__Hansen, MEB
Tanzania__Burunge__Hansen, MEB
Cameroon__Pygmy__Morton, ER
Cameroon__Bantu__Morton, ER
Central African Republic__Bantu__Gomez, A
Central African Republic__BaAka__Gomez, A
Malawi__Malawi__Yatsunenko, T
Burkina Faso__Burkina Faso__De Filippo, C
Botswana__San__Hansen, MEB
Botswana__Herero__Hansen, MEB
Botswana__Bantu__Hansen, MEB
Papua New Guinea__Nauna Village (Sausi)__Martinez, I
Papua New Guinea__Asaro Valley__Martinez, I
Peru__Tunapuco__ObregonTito, AJ
Venezuela__Yanomami__Clemente, JC
Venezuela__Guahibo__Yatsunenko, T
China__China__Nakayama, J
Japan__Japan__Nakayama, J
Australia__Australia__AGP_Australia
Italy__Italy__Schnorr, SL
Italy__Italy__De Filippo, C
Ireland__Ireland__Claesson, MJ
United Kingdom__United Kingdom__AGP_United_Kingdom
Canada__Canada__AGP_Canada
USA__USA__Yatsunenko, T
USA__USA__HMP v35
USA__USA__HMP v13
USA__USA__AGP_USA
USA__Philadelphia__Wu GD; Minot S; Ni J
USA__Norman__ObregonTito, AJ
−5.0 −4.5 −4.0 −3.5 −3.0 −2.5 −2.0 −1.5 −1.0
Log Rel. Abund. Spirochaetes

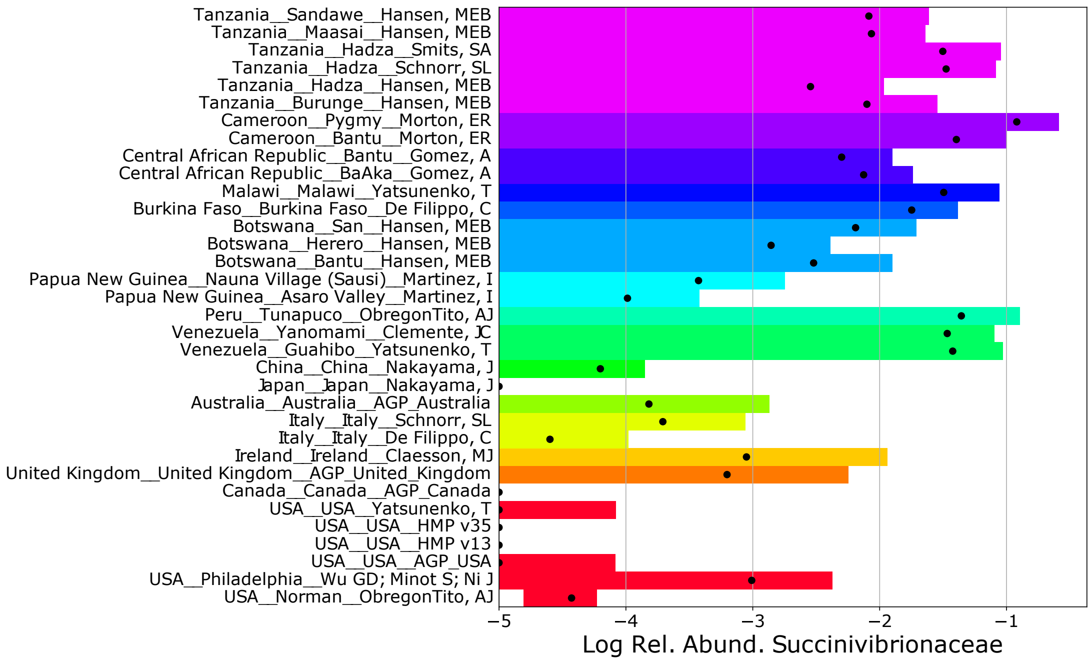

Tanzania__Sandawe__Hansen, MEB
Tanzania__Maasai__Hansen, MEB
Tanzania__Hadza__Smits, SA
Tanzania__Hadza__Schnorr, SL
Tanzania__Hadza__Hansen, MEB
Tanzania__Burunge__Hansen, MEB
Cameroon__Pygmy__Morton, ER
Cameroon__Bantu__Morton, ER
Central African Republic__Bantu__Gomez, A
Central African Republic__BaAka__Gomez, A
Malawi__Malawi__Yatsunenko, T
Burkina Faso__Burkina Faso__De Filippo, C
Botswana__San__Hansen, MEB
Botswana__Herero__Hansen, MEB
Botswana__Bantu__Hansen, MEB
Papua New Guinea__Nauna Village (Sausi)__Martinez, I
Papua New Guinea__Asaro Valley__Martinez, I
Peru__Tunapuco__ObregonTito, AJ
Venezuela__Yanomami__Clemente, JC
Venezuela__Guahibo__Yatsunenko, T
China__China__Nakayama, J
Japan__Japan__Nakayama, J
Australia__Australia__AGP_Australia
Italy__Italy__Schnorr, SL
Italy__Italy__De Filippo, C
Ireland__Ireland__Claesson, MJ
United Kingdom__United Kingdom__AGP_United_Kingdom
Canada__Canada__AGP_Canada
USA__USA__Yatsunenko, T
USA__USA__HMP v35
USA__USA__HMP v13
USA__USA__AGP_USA
USA__Philadelphia__Wu GD; Minot S; Ni J
USA__Norman__ObregonTito, AJ
−5 −4 −3 −2 −1
Log Rel. Abund. Succinivibrionaceae

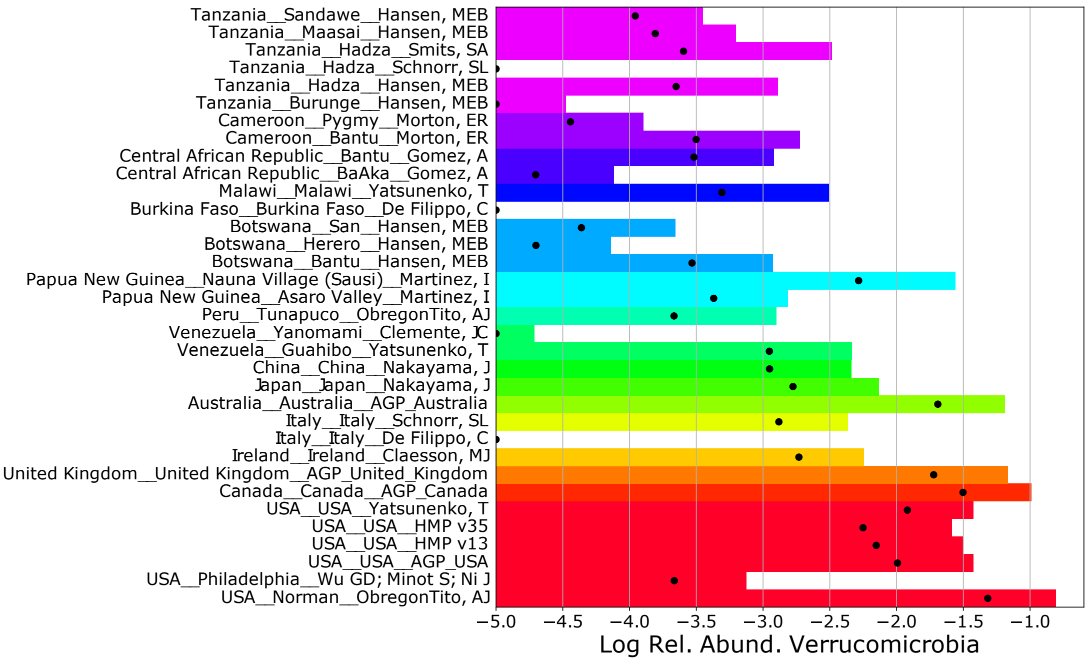

Tanzania__Sandawe__Hansen, MEB
Tanzania__Maasai__Hansen, MEB
Tanzania__Hadza__Smits, SA
Tanzania__Hadza__Schnorr, SL
Tanzania__Hadza__Hansen, MEB
Tanzania__Burunge__Hansen, MEB
Cameroon__Pygmy__Morton, ER
Cameroon__Bantu__Morton, ER
Central African Republic__Bantu__Gomez, A
Central African Republic__BaAka__Gomez, A
Malawi__Malawi__Yatsunenko, T
Burkina Faso__Burkina Faso__De Filippo, C
Botswana__San__Hansen, MEB
Botswana__Herero__Hansen, MEB
Botswana__Bantu__Hansen, MEB
Papua New Guinea__Nauna Village (Sausi)__Martinez, I
Papua New Guinea__Asaro Valley__Martinez, I
Peru__Tunapuco__ObregonTito, AJ
Venezuela__Yanomami__Clemente, JC
Venezuela__Guahibo__Yatsunenko, T
China__China__Nakayama, J
Japan__Japan__Nakayama, J
Australia__Australia__AGP_Australia
Italy__Italy__Schnorr, SL
Italy__Italy__De Filippo, C
Ireland__Ireland__Claesson, MJ
United Kingdom__United Kingdom__AGP_United_Kingdom
Canada__Canada__AGP_Canada
USA__USA__Yatsunenko, T
USA__USA__HMP v35
USA__USA__HMP v13
USA__USA__AGP_USA
USA__Philadelphia__Wu GD; Minot S; Ni J
USA__Norman__ObregonTito, AJ
−5.0
−4.5
−4.0
−3.5
−3.0
−2.5
−2.0
−1.5
−1.0
Log Rel. Abund. Verrucomicrobia
